# Community Health Worker Feedback on an mHealth Intervention for Hypertension in Rural Guatemala: Mixed Methods Formative Study

**DOI:** 10.2196/75471

**Published:** 2026-04-17

**Authors:** Alvaro Bermudez-Cañete, Pablo Nuñez-Perez, Sean Duffy, Alejandro Chavez, Ian Stanley Arthur, Rafael Tun, Yoselin Letona Lopez, Celina Perez, Taryn McGinn Valley

**Affiliations:** 1 Department of Bioengineering Stanford University Stanford, CA United States; 2 Department of Family Medicine and Community Health University of Wisconsin-Madison Madison, WI United States; 3 School of Medicine University of California San Francisco San Francisco, CA United States; 4 Hospital Obras Sociales Monseñor Gregorio Schaffer San Lucas Mission San Lucas Guatemala; 5 Department of Anthropology; Medical Scientist Training Program, School of Medicine and Public Health University of Wisconsin-Madison Madison, WI United States

**Keywords:** hypertension management, mHealth app, mobile health, community health workers, Guatemala, digital health, rural health, noncommunicable diseases

## Abstract

**Background:**

Hypertension remains a leading global health challenge, particularly in low- and middle-income countries (LMICs), where limited health care infrastructure and resources restrict effective management. Community health workers (CHWs) are critical in delivering care in these settings, and when equipped with mobile health (mHealth) apps, they can greatly enhance chronic disease management. Involving CHWs in the design and development at all stages is essential for the success of such programs. However, relatively little research discusses CHW feedback on mHealth interventions.

**Objective:**

This study aims to evaluate CHW feedback on a hypertension program using a novel tablet-based mHealth tool designed for CHW hypertension diagnosis and management in rural Guatemala.

**Methods:**

We conducted a mixed-methods analysis as part of a pilot study in San Lucas Tolimán, Guatemala, involving 6 CHWs over a 6-month period. Quantitative data were collected using the System Usability Scale and Likert-scale surveys before and after study completion. Qualitative data were gathered through written surveys and focus group interviews conducted in Spanish by bilingual team members. These methods assessed the app’s ease of use, workflow integration, and cultural appropriateness. CHWs provided detailed perspectives on technical challenges, training adequacy, and patient engagement, which guided iterative refinements to both the mHealth app and the hypertension management program.

**Results:**

The mHealth app was generally well-received. Average System Usability Scale scores exceeded 70, surpassing established usability thresholds. Likert scale data revealed CHWs found the app to be useful and easy to use, but identified training protocols as areas for improvement. Qualitative analysis of focus groups and written surveys revealed 3 dominant themes. First, CHWs identified practical short-term needs, including slower and more comprehensive training sessions, simplified medication dosing regimens to reduce pill burden, and streamlined survey questions to shorten patient visit times. Second, CHWs raised larger structural concerns, including retention challenges related to financial compensation and misalignment between required clinical data collection and the cultural appropriateness of certain app questions. Third, CHWs highlighted program benefits, including improved patient care and hypertension management, empowerment through educational tools, and increased pride and community trust associated with the program.

**Conclusions:**

Our findings suggest that iteratively integrating user feedback into the development of mHealth interventions is key to improve usability, cultural appropriateness, and overall effectiveness of chronic disease management in resource-constrained settings. Due to the small number of CHW participants, as well as a reliance on self-reported perceptions, these findings should be interpreted as exploratory and hypothesis-generating rather than generalizable. This study contributes to the growing literature on mHealth apps for noncommunicable diseases in LMICs and provides insights into CHW experiences. Addressing the technical barriers and systemic challenges identified in this study can help improve future implementations of mHealth-enabled chronic disease programs in LMICs.

## Introduction

Hypertension is the leading risk factor for death globally, and a major cause of premature death worldwide [1]. Of the estimated 1.4 billion adults worldwide living with hypertension, two-thirds live in low- and middle-income countries (LMICs) [2]. Despite the high prevalence of hypertension, estimates suggest that globally 44% of adults with hypertension are unaware of their condition [2]. Moreover, only 44% are diagnosed and treated, with just 23% having hypertension that qualifies as controlled [2]. Awareness, treatment, and control rates are notably higher in urban than rural areas in LMICs [3].

Limited health care infrastructure, low physician-to-patient ratios, and lack of access to medicines are among the key challenges disproportionately faced by LMICs, further contributing to the burden of hypertension [4]. To help address these health care inequities, community health worker (CHW) programs have been widely implemented, often enhancing health system capacity for both acute and chronic conditions by supporting tasks traditionally performed by physicians [5,6]. The use of mobile health (mHealth) technologies by CHWs has emerged as promising for health care delivery in LMICs across various contexts, including the management of noncommunicable diseases [7-10].

Our team developed a novel mHealth app as part of a larger hypertension program aimed at facilitating the diagnosis and management of hypertension by CHWs in San Lucas Tolimán, a rural municipality in Guatemala. The details of the program, training, and app’s feasibility have been discussed, tested, and published previously [11], but to provide context for this study, here is a summary. The program equips CHWs with a tablet running the app using a platform called CommCare (Dimagi), enabling them to enroll patients, record medical and lifestyle history, and track relevant blood pressure measurements. At the end of the patient visit, the app provides both diagnostic guidance and hypertensive treatment recommendations (Multimedia Appendix 1). An ongoing clinical trial (ClinicalTrials.gov NCT06444308) seeks to compare the CHW intervention incorporating the app with that of a physician, the traditionally accepted standard of care [12].

This study focuses on the structured feedback system used to iteratively evaluate and refine the implementation of this hypertension program. In this phase of the study, 6 trained CHWs helped implement the pilot as members of the study team; they also provided feedback, which we analyzed here. We present our experience using multimodal feedback to identify implementation barriers, training gaps, and workflow inefficiencies. We accomplished this by collecting quantitative and qualitative feedback from CHWs through System Usability Surveys (SUS), written surveys, and focus group discussions. By centering CHWs as active contributors to the program, this study highlights a feedback-driven approach that can inform the design and adaptation of mHealth-supported chronic disease programs in resource-limited settings.

In LMICs, where access to health care technology is still evolving, adoption and implementation challenges are significant [13], and studies focusing on how implementation challenges are identified, communicated, and addressed remain limited [14,15]. Iterative feedback at all steps of the design and implementation process is crucial for ensuring that the intervention is functional, culturally sensitive, and adaptable to the specific needs of the community. This study addresses this gap by evaluating the feedback of CHWs in rural Guatemala on the use of one mHealth app.

## Methods

### Study Design

Our group recently conducted a pilot study to evaluate the feasibility and effectiveness of a CHW-led, mHealth-supported hypertension management program in rural Guatemala. The mHealth app used for the study was developed through regular consultations with CHWs and community members to ensure that the app considered challenges commonly faced in rural health care delivery [16]. Our research team consists of male and female physicians, medical students, graduate students, undergraduate students, and health care administrators. Some team members live in Guatemala, and others in the United States. Approximately half of the team members were born and continue to live in rural Guatemala. All members speak Spanish; 2 learned Spanish as children and are fluent speakers, while the rest of the team are native Spanish speakers. About one-half of the team speaks Kaqchikel, the Indigenous Mayan language spoken by many in San Lucas Tolimán.

Data collection for the study presented here focused on the app’s ease of use, workflow challenges, and the overall impact of the program on the CHWs’ ability to provide care, as these factors are key facilitators for the sustainability of digital health interventions in LMICs [17]. Our goal was to identify areas of success and where improvement was needed in the app workflow, study protocol, and overall hypertension program. In accordance with this framework, one component of this study focused on technical usability in introducing CHWs to digital tools largely unfamiliar to them. The other examined how well the mHealth solution aligned with local practices, cultural norms, and its impact on CHW-patient interactions, which are critical for cultural and contextual adaptation [18].

### Study Setting

The study took place in San Lucas Tolimán, a municipality in Guatemala’s Western Highlands with a population of just over 30,000 people, most of whom identify as Kaqchikel Maya. Approximately half of the residents reside in the 19 rural villages that surround the main town [19]. Guatemala has the second-highest poverty rate in Latin America, with rural regions disproportionately affected. It is estimated that 80% of Guatemala’s rural population lives in such conditions [20]. Socioeconomic barriers like inequitable investments in infrastructure, education, and sanitation contribute to significant health disparities. Additionally, many villages are remote with few transportation options, further restricting access to formal health care services [21]. Notably, many disparities stem from histories of colonialism and genocide, including post–civil war divestment in rural infrastructure and health care, leaving many Indigenous communities in rural Guatemala with inadequate services and economic opportunities [22].

### Participants

The study examined feedback from 6 CHWs, known locally as *promotores de salud*. They are a part of a health program managed by the San Lucas Mission, a nonprofit organization with which we partner that provides health care and social services to local residents.

For the purposes of this feedback study, participants were drawn from the cohort of CHWs already enrolled in the larger pilot program. Their recruitment for the pilot occurred in-person during routine program meetings and was conducted by members of the study team in collaboration with San Lucas Mission leadership. From this group, we used purposive sampling to invite CHWs to participate in the current feedback study. All 6 eligible CHWs agreed to participate in the additional feedback activities.

During the pilot, the 6 CHWs used our mHealth app during routine patient visits supporting hypertensive care. This participation informed the perspectives discussed in this study. All participants completed a structured training program to partake in the pilot study, including instruction in hypertension management and app use (Multimedia Appendix 2). This training ensured a mutual baseline of technical competency, which allowed the feedback collected to focus on experiential, cultural, and structural considerations rather than basic operational challenges.

### Data Collection

Data collection aimed to capture both the technical and cultural dimensions of the program. This was accomplished using three complementary methods:

1. SUS: usability was assessed using the SUS, a validated 10-item questionnaire that measures perceived ease of use and system functionality (Multimedia Appendix 3). Widely used in technology and health care research, the SUS helps evaluate user experience across various digital tools, from mobile apps to medical devices [23]. Each item was rated on a 5-point Likert scale (1=Strongly Disagree and 5=Strongly Agree). Negatively worded SUS items (Q2, Q4, Q6, Q8, and Q10) were reverse-coded prior to score calculation so that higher values consistently reflected better usability. Five CHWs completed the survey, administered at month 0 and month 6, to evaluate changes in CHWs’ perceptions of the mHealth app over time.
2. Written surveys: 5 CHWs completed written surveys with open-ended questions to provide qualitative feedback on the app’s strengths, challenges, and areas for improvement (Multimedia Appendix 3).
3. Focus group interviews: two 1-hour focus group interviews were conducted, one at the end of the pilot study and another 4 months post study, to gather in-depth insights on 6 CHWs’ experiences. These discussions were conducted in Spanish, led by 2 (first group) or 1 (second group) of 3 bilingual team members. Interviewers used a guide to ensure that key topics were discussed, including usability, training adequacy, and broader program challenges (Multimedia Appendix 4).

Qualitative data saturation was reached after written surveys and 2 focus group interviews. Many themes were repeated across surveys and focus groups, and these conversations ended with time to spare. Additionally, we had interviewed and requested surveys from every CHW who participated as a study member in this pilot study.

### Data Analysis

For our limited quantitative analysis, SUS scores were analyzed following the method described by Bangor et al [24]. Scores on the 5-point Likert scale were summed and multiplied by 2.5 to scale the final SUS score to a 0-100 range. Given the low sample size of the study (n=5), we did not conduct statistical analysis by demographics.

For the qualitative data, focus group interviews were audio-recorded in Spanish, transcribed verbatim, and translated into English by 2 bilingual members of the research team. Both members used a memoing approach, annotating the document directly before translating to English. This was completed independently, and then discrepancies were resolved to minimize interpretive bias. Both translators have experience working with CHWs directly, which helped them interpret local terminology. These 2 members did not conduct the interviews themselves, potentially reducing the possibility of translation biases due to interviewer-participant dynamics. Still, we understand that any translation, in this case from Kaqchikel-influenced Spanish to English, carries the risk of shifting nuance, and this may affect interpretation.

We reported the qualitative components of this study in accordance with the COREQ (Consolidated Criteria for Reporting Qualitative Research) checklist, which was completed and supplied for editorial review. Analyses were made using an inductive coding approach. This meant that, rather than imposing codes from a predefined framework, we allowed CHWs’ language to shape the analytical results. This approach is well-suited to exploratory qualitative analysis, where explanations are derived from the data, rather than testing preexisting theories [25]. We identified themes primarily based on immediate usefulness to our study implementation. These were organized into 3 categories: “Practical short-term needs,” “Larger structural concerns,” and “Program Benefits.” We then selected specific participant quotes to illustrate key insights.

### Ethical Considerations

#### Ethics Approval

This set of studies was approved by the University of Wisconsin Institutional Review Board (ID # 2022-0794) as well as the San Lucas Healthcare Committee, an interdisciplinary, international group that provides local ethics oversight to health care projects. The overarching hypertension program, including earlier feasibility phases, was also registered with ClinicalTrials.gov (ID NCT05479097). This substudy was exempt from additional institutional review board review because the CHW study participants were, in fact, researchers on the study themselves.

#### Informed Consent

CHWs signed documentation to take part in the overarching hypertension pilot study as study team members, and these specific CHWs provided verbal consent to participate in focus groups, written surveys, and usability assessments included in this substudy.

#### Privacy and Confidentiality

Since the CHWs were actively involved in the pilot study, complete anonymity during data collection was not feasible. However, all qualitative and quantitative data were deidentified prior to analysis, and no personally identifiable information was included in analytic datasets or reported in the results. Audio recordings and transcripts were stored on secure, password-protected servers accessible only to authorized study personnel.

#### Participant Compensation

CHWs did not receive additional compensation specifically for participation in this feedback substudy beyond their standard stipends for program-related activities at the time of the pilot.

## Results

### Participant Demographics

In total, 6 CHWs participated in the pilot study. At the onset of the study, the 6 participating CHWs had an average age of 42.2 (SD 8.09) years. Five were female, while 1 was male. Four had completed elementary school, one had attended middle school, and one had obtained an associate’s degree. All CHWs reported using a smartphone daily.

### System Usability Survey Scoring

At the beginning of the pilot, the average system usability score (SUS) was 72.5 out of 100, which decreased marginally to 71.25 by the end of the 6-month period. Specific results of the survey are seen in Table 1.

**Table 1:** Analysis of System Usability Scale responses before and after the pilot study.

Items	Questions	Before, mean (SD)	After, mean (SD)
Q1	I think I’d like to use the app frequently.	4.75 (0.43)	5 (0)
Q2	The app is unnecessarily complicated.	5 (0)	4 (0)
Q3	I find the app easy to use.	4 (1.73)	4.25 (0.43)
Q4	I believe I’d need help from a technician to use the app.	2.50 (1.50)	3.25 (1.30)
Q5	The different functions of the app are well integrated.	4.50 (0.50)	3.25 (0.83)
Q6	I find the app confusing.	5 (0)	4.25 (0.83)
Q7	I imagine most people would learn to use this app very quickly.	2.25 (1.30)	3.50 (1.12)
Q8	I find the app very difficult to use.	4.75 (0.43)	4.75 (0.43)
Q9	I feel confident using the app.	4.25 (0.83)	5 (0)
Q10	I needed to learn many things about this app before I could use it.	1.50 (0.50)	2 (1.23)

### Categorized Qualitative Feedback

We categorized qualitative data from written surveys into 3 main themes: practical short-term needs, larger structural concerns, and program benefits. We organized the themes in this way to be the most useful in ongoing project implementation, reflecting the pragmatic aims of the work, a method grounded in established qualitative research practices [25]. Table 2 provides a summary of these key insights, offering concrete examples of CHWs’ experiences with the mHealth app and highlighting specific areas for improvement and success.

**Table 2:** Key insights for improvement from community health workers’ focus groups.

Categories	Quotes	Interpretation
Practical short-term needs	“Patients say they need to take too many pills, but we feel like the medication doses are just too low.” “Trainings to learn about hypertension could be taught a bit more slowly.” “Questions in the app seem to repeat. These repetitions make using the app quite slow.”	Highlights tension between algorithm dosing and actual pill burden. Suggests either needing a clearer CHW^a^-facing explanation of dosing rationale or adjusting pill dosages. Indicates that training pace may be a barrier to effective adoption. Emphasizes the importance of longitudinal training models. Reveals app workflow inefficiencies. Suggests shortening survey questions to shorten visit length and improve patient and CHW satisfaction.
Larger structural concerns	“For us working here, is there going to be more compensation?” “It is often hard because in our culture we are not used to asking women how many times they urinate; this is not normal for us Indigenous people.” “It may be common for women in other countries to drink [alcohol] often, but here in Guatemala they don’t tend to, and if you ask them, they get offended.”	Signals a potential retention and motivation issue. This challenge is a common barrier to long-term CHW program implementation, where compensation is often limited due to financial constraints. Suggests shifting financial priority toward workers. Demonstrates a cultural mismatch between standardized clinical questions and local norms. Indicates the necessity of culturally adapted questions, although many times it is difficult to align, since changes or omissions may affect the validity and reliability of clinical trial outcome measures.
Program benefits	“The program benefits many people in our communities since receiving care is often hard to get, but they can with the program.” “Our educational flip book helps make patients aware, specifically the objectives of the program and causes of hypertension.” “The patients look for us because they trust us. They know we can help them and that we are here for them.”	Emphasizes the importance of improved access to care, reinforcing program value and motivation. Identifies educational material as key for facilitating patient engagement and understanding. Highlights trust as a central mechanism through which CHWs enable hypertension management.

^a^CHW: community health worker.

### Practical Short-Term Needs

CHWs identified several practical and readily implementable strategies to improve the management of hypertension in rural settings.

CHWs reported that many patients were frustrated by the need to take multiple pills daily, which they perceived as increasing regimen complexity and undermining adherence. To address this, CHWs recommended reducing pill burden by using higher-dose formulations when clinically appropriate, emphasizing regimen simplification as a practical strategy to support adherence. CHWs also emphasized the need for greater transparency in treatment protocols, explaining they wanted to better understand the reasoning behind the app's recommendations and workflow design.

Several CHWs acknowledged that fasting for laboratory testing—required twice over the pilot period, at enrollment and at 6 months—helps ensure reliable results for patient care purposes and scientific rigor, even though they felt it could inconvenience patients and potentially reduce attendance. In addition, CHWs advocated for a slower, more comprehensive training process; while they stated appreciating their training and education on hypertension, they said that additional reinforcement could deepen their understanding and improve patient management.

Finally, CHWs mentioned multiple times that the app contained survey questions they found both burdensome and redundant. CHWs expressed awareness, due to the use of this and previous apps during multiple phases of development, that additional questions, on average, made mHealth apps work more slowly. They acknowledged the importance of being thorough and adhering to guidelines for diagnosis and treatment. However, they suggested that reducing the number of questions would make the process easier for patients and CHWs themselves.

### Larger Structural Concerns

CHWs raised challenges that we categorized as more difficult to implement due to cultural and structural barriers. One significant issue was the cultural sensitivity of certain questions within the app, such as those related to alcohol consumption and urination frequency. CHWs shared that these topics are rarely discussed in their communities, and that they sensed that these questions often made patients feel uncomfortable (Table 2).

CHWs also stressed the need for better compensation and incentives for their own participation, as they felt the financial support for their work at the time was insufficient, given the demands of their role. They further highlighted the difficulty of reaching patients in remote or difficult-to-access areas.

### Program Benefits

#### Enhanced Patient Care and Hypertension Management

Although the focus group interview guide focused on areas of potential improvement, CHWs also shared positive feedback on the program. They emphasized its positive impact on patient care and how it increased their ability to deliver critical health care services to their communities. They noted that, in their opinions, the mHealth app itself contributed to improved hypertension management, enabling patients to access care that had previously been difficult to obtain.

#### Empowerment Through Educational Tools

CHWs approved of the program tools for patient education. They found that the mHealth app, along with supplementary materials such as several educational flip books, helped them educate patients on hypertension. These resources helped patients understand their condition and increased CHWs’ own medical knowledge. CHWs explained how these educational tools made them feel more confident, particularly when explaining medication adherence and lifestyle changes to patients.

### Pride and Community Trust

Finally, CHWs expressed pride and satisfaction in their work, recognizing their contributions to improving health and well-being in their communities. They valued the relationships they built with community members, specifically the trust they described as having fostered through their consistent presence and support. Their dedication to continuous learning and skill development further reinforced their motivation, as they saw tangible improvements in both their abilities and the well-being of their patients.

## Discussion

### Summary of Principal Findings

This study aimed to characterize CHW feedback on the usability, cultural fit, and implementation challenges of a tablet-based mHealth app supporting hypertension care in rural Guatemala. Analysis of quantitative and qualitative feedback indicated that CHWs generally perceived the app as usable and acceptable within their routine work and reported that the program supported hypertension care in communities with limited access to formal health services.

In addition to these perceived benefits, CHWs identified several implementation challenges and areas for improvement. These included workflow inefficiencies driven by survey burden and repetitive questions that lengthened patient visits; the need for more comprehensive and slower-paced training, including additional practice scenarios and guidance on how to explain hypertension and treatment recommendations to patients; and medication-related concerns, particularly the complexity of pill dosing regimens and perceptions of pill burden. CHWs raised concerns about the cultural appropriateness of certain intake questions. They also identified broader structural constraints, including compensation and retention challenges. At the same time, CHWs described meaningful program benefits, including increased confidence in delivering hypertension care, enhanced capacity to educate patients, empowerment through educational tools, and strengthened trust and pride within their communities.

### Interpretation and Implications

Together, these findings highlight both the feasibility of CHW-led, mHealth-supported hypertension care in this setting and the importance of incorporating frontline feedback to guide ongoing program refinement. Encouraging CHWs to critically examine the intervention secured insights from its primary users, which informed iterative improvements to the program and app.

### Likert Scale Usability Surveys

Likert scale data showed overall SUS scores averaging above 70, suggesting the app’s general acceptability and functionality in one town in rural Guatemala in this small pilot sample. These scores surpassed the threshold of acceptability as defined by Bangor et al [24], indicating that the app was generally perceived as well-received by the CHWs and aligned with their capabilities and expectations.

CHWs who took both pre and post surveys rated different usability questions on the app differently. Two questions with relatively low scores included Q4 and Q10, related to potential technician help for app use and prior knowledge needed before using the app, respectively. Given the small sample size, these patterns are interpreted descriptively rather than as statistically meaningful differences. This result suggests areas for further attention, particularly regarding technical support and adequate training when implementing an app with complicated clinical functions such as ours.

Study team members felt that CHW participants’ comfort in expressing their opinions increased over the pilot period. We observed more extensive written responses on the 6-month surveys, attributed to both deeper CHW understanding of the app’s functionality and workflow improvements, and to increased trust that led to more open communication and honest, constructive feedback.

### Cultural and Contextual Adaptation

Program and app development occurred with continuous feedback from CHW coordinators, building on over 5 years of prior work in these same communities with similar mHealth apps. Yet, despite these efforts, CHWs perceived some cultural barriers and discomfort in using the novel tool within this specific program context, raising the key question of why these issues persisted.

Previous scholars have highlighted the importance of cultural sensitivity in designing and implementing global health interventions, including mHealth tools [26-28]. In our study, CHWs highlighted that certain questions, such as those related to alcohol consumption and urination frequency, were uncomfortable to ask and for patients to answer in their communities. CHWs explained explicitly that in their own communities, social norms and health conversations may differ from places where other team members are from. Our findings build upon established literature by illustrating, within this specific setting, that cultural alignment enhances usability and trust between CHWs and patients, an important factor for the long-term success of mHealth tools alongside their technical functionality. Our study aligns with conclusions from those who emphasize the importance of iterative cultural adaptation in the design process [29-31]. Adapting mHealth tools is a dynamic process: once new content is introduced, unanticipated challenges may arise. Ongoing dialogue and iterative tool improvement can help apps like ours remain culturally appropriate for end users over time.

### Training

While the mHealth tool was designed to minimize training burden by offering user-friendly interfaces and decision-support tools, CHWs expressed a need for deeper training on both hypertension management and the app’s use. This highlights a common challenge in digital health interventions: while such tools can streamline tasks, they require strong training programs to ensure users feel capable and confident [32].

In our specific context, CHWs have decades of expert knowledge about their communities, but some struggle with literacy. Our own in-house training may be some of the first formal instructions they have received on using digital tools. At the same time, all CHWs use smartphones, so many have intuitive understandings of technology use that they bring to their mHealth work. Additionally, all CHWs who participated in the pilot study had worked with earlier mHealth tools from our team [33-35]. To address diverse training needs, we used iterative, case-by-case reinforcement, particularly for CHWs with literacy challenges or gaps in understanding hypertension management. Training alternated among group discussions, one-on-one support, and practical case-based scenarios tailored to individual strengths. The feedback CHWs provided gave us better insight – training should go slower, cover more scenarios, and train CHWs specifically on how to explain health concerns to patients. This aligns with our group’s participatory experience that traditional, classroom-style didactics were less well received than one-on-one or small-group practical and case-based learning.

### Workflow Optimization

A key distinction between research protocols and routine clinical care helps contextualize some CHW feedback on the app’s perceived slowness. In our research context, the decision support system was designed to capture additional data for our own research purposes—data that, while valuable, is not strictly necessary for everyday clinical care. This included survey questions that CHWs found repetitive and burdensome but that were required as part of the study protocol. Rather than simply eliminating these questions, we refined technical features to increase app speed: we restructured the forms, reduced redundancies when appropriate, used faster-processing tablets, and divided forms into multiple sections that still operate as a single cohesive interface for the CHWs. These changes have significantly improved performance while preserving certain research elements that differentiate our study from routine clinical practice [36]. This highlights a broader tension between the data requirements of research protocols and the efficiency in digital health research.

### Compensation and Structural Barriers

The study also revealed structural challenges beyond the usability of the mHealth tool, specifically around compensation for CHWs within the program. Many CHWs at the time of the study were essentially unpaid volunteers, who only received a small stipend on days they were asked to work. This lack of reliable financial support raised significant concerns about the sustainability of CHW-led health programs. Notably, after this study was completed but before submission of the manuscript, the local nongovernmental organization (NGO) and its American funders overhauled CHW compensation to achieve parity with other hospital employees. This process, while it involved administrative challenges outside of the scope of this study, introduced reliable income and benefits to a cadre of workers that had never experienced this type of labor support. In a country like Guatemala, where most employment does not meet this level of formality or security, this represented a meaningful programmatic change. Indeed, the program team’s efforts to support the employment overhaul were informed by CHW feedback we present in this study. Our experiences working with CHWs in LMICs yield an important lesson for researchers: while mHealth technologies can support health care delivery, technologies’ successes are closely tied to the well-being and motivation of the workers using them. Providing fair compensation is important to ensure the long-term viability and scalability of CHW programs in LMICs. Therefore, future interventions should focus not only on technological aspects but also on providing adequate compensation, training, and institutional support to create sustainable health care programs in low-resource settings.

### Limitations

The small sample size of 6 CHWs and the relatively short 6-month duration of the pilot limit the extent to which these results can be applied to other settings. We nonetheless feel our findings are useful and contribute to the state of knowledge in the field. We benefited from the capacity to quickly evaluate and iterate on our initial results, including those presented in this study.

The SUS, as a measure of usability, provides valuable insights but cannot capture the full complexity of user experiences. Since only 5 CHWs completed both the pre- and post surveys, our SUS analysis was additionally limited. Future studies from our team could benefit from a more comprehensive quantitative evaluation framework that incorporates a wider range of usability metrics and long-term follow-up data. Additional constraints in our limited quantitative data include the ambiguity of Likert-scale scoring, which we are exploring in future research, and inconsistencies in how users interpret and rate their experiences [37]. The assumption that ordinal SUS scores behave linearly can also introduce variability, complicating direct comparisons.

Additionally, qualitative findings are subject to limitations inherent to translation. Although focus groups were conducted in Spanish and translated by bilingual team members familiar with local terminology, participants’ speech was often influenced by Kaqchikel, and translation into English may have shifted nuance or meaning, potentially affecting interpretation. Just like the quantitative data, our qualitative data were pulled from a small sample, and our interview guide was quite programmatic, not designed to pull de novo theory regarding health care delivery. Nonetheless, we believe our qualitative data was rich and helpful in our project's formative phase.

The scalability of our findings is limited by the nature of our partnership with a local NGO rather than working within the national health system. Our work with the NGO allowed us to develop a more nuanced, adaptable tool. However, we recognize that this model is not directly transferable to national health systems, which often operate on larger scales where reaching research agreements can take years, limiting the capacity for quick iteration. Nonetheless, we demonstrate how learning directly from CHWs can inform the design of mHealth tools that can later be modified to fit broader contexts.

### Conclusions

The implications of this study extend beyond rural Guatemala. The methods outlined in this study provide a context-informed model for the evaluation and iterative refinement of similar mHealth solutions in other LMIC settings, including at the level of small pilot studies. More broadly, this work demonstrates the value of formative, CHW-centered evaluation in identifying factors such as training, workflow, cultural alignment, and structural supports that shape the real-world implementation of digital health interventions. Researchers developing novel interventions like mHealth tools should ensure their use is driven by the specific needs and expectations of the communities in which they work. By integrating community feedback and attending to cultural sensitivity during an iterative design process, we provide practical guidance for how future mHealth initiatives can enhance usability and impact.

## Data Availability

The data generated during this study are not publicly available due to ethical and confidentiality considerations related to the small sample size and the inclusion of qualitative interview data. Requests for access to deidentified data can be directed to the corresponding author and will be considered on a case-by-case basis.

## Appendix

Multimedia Appendix 1 Diagram of the mHealth Application Workflow.

Multimedia Appendix 2 Community health worker training PowerPoint.

Multimedia Appendix 3 Likert scale survey template and written survey questions.

Multimedia Appendix 4 Focus group interview question guide.

## Abbreviations

CHW: community health worker
COREQ: Consolidated Criteria for Reporting Qualitative Research
LMIC: low- and middle-income country
mHealth: mobile health
NGO: nongovernmental organization
SUS: System Usability Scale
