# Supplementary figures and images for: Community Health Worker Feedback on an mHealth Intervention for Hypertension in Rural Guatemala: Mixed Methods Formative Study
Source: JMIR Form Res. 2026 Apr 17;10:e75471. doi: 10.2196/75471 (PMC13135166; doi:10.2196/75471)

## Diagram of the mHealth Application Workflow

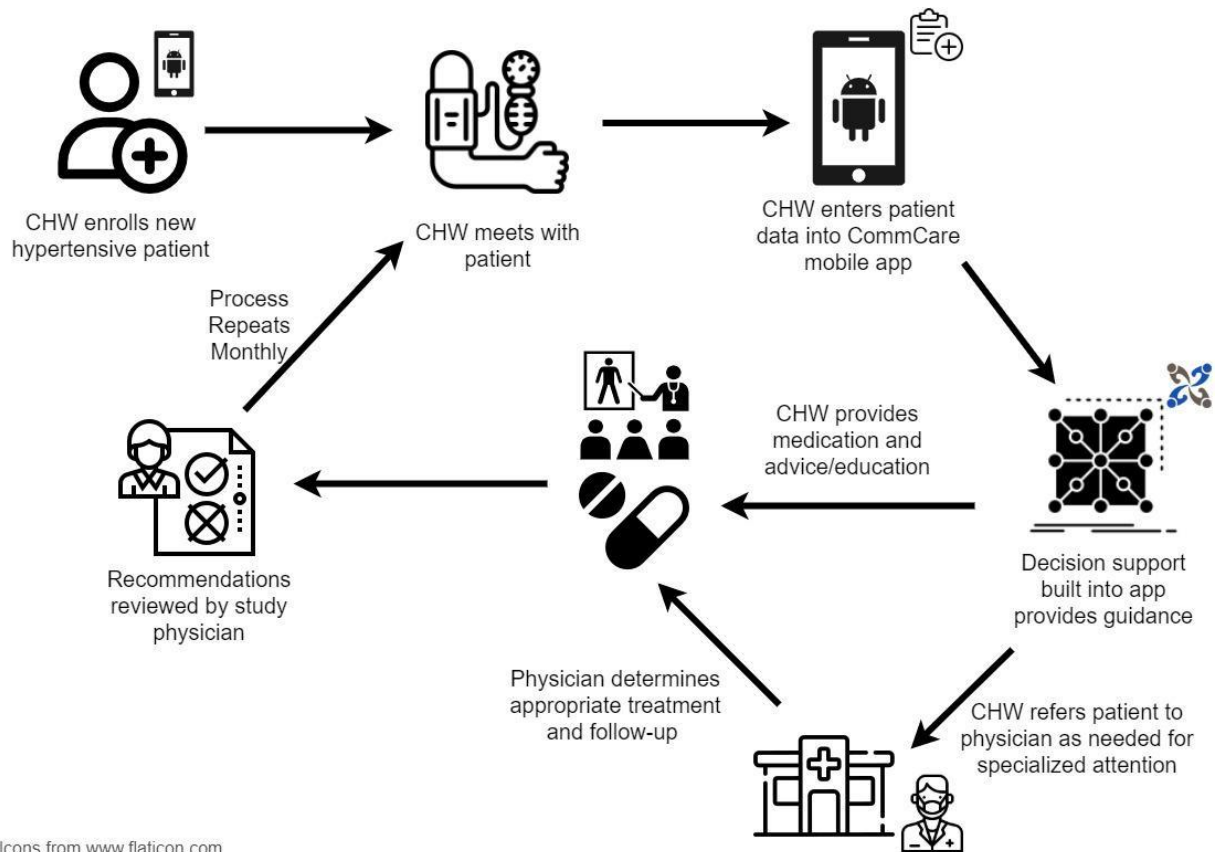

Icons from [www.flaticon.com](http://www.flaticon.com)

Supplement: Multimedia Appendix 1 [file formative_v10i1e75471_app1.pdf]
