# Supplementary material for: Community Health Worker Feedback on an mHealth Intervention for Hypertension in Rural Guatemala: Mixed Methods Formative Study
Source: JMIR Form Res. 2026 Apr 17;10:e75471. doi: 10.2196/75471 (PMC13135166; doi:10.2196/75471)
Supplement: Multimedia Appendix 2 [file formative_v10i1e75471_app2.pdf]

# Introducción a la Iniciativa de Hipertensión

Valerie Aguilar, Estudiante de medicina
Juan Aguirre, Estudiante de medicina
Sean Duffy, MD, MPH
Universidad de Wisconsin

# Agenda

Jueves, 7/21

- Básico de hipertensión
- Complicaciones
- Derrame cerebral
- Prevencion

Miercoles, 7/27

- Medicamentos y efectos secundarios
- Laboratorios
- Comorbilidades
- Practicar medidas de presión
- Práctica de aplicación

# Introducción a la iniciativa de hipertensión en las comunidades rurales de San Lucas Tolimán

Department of Family Medicine and Community Health
UNIVERSITY OF WISCONSIN
SCHOOL OF MEDICINE AND PUBLIC HEALTH

NIH Fogarty International Center
*Advancing Science for Global Health*

MISIÓN
SOLIDARIDAD
DIGNIDAD
BIEN COMÚN
SUBSIDIARIEDAD
San Lucas Tolimán
¡¡¡Juntos Podemos!!!

HOSPITAL
OBRAS SOCIALES
"Mons. Gregorio Schaffer"

# Lo Básico de la Hipertensión

# El sistema circulatorio

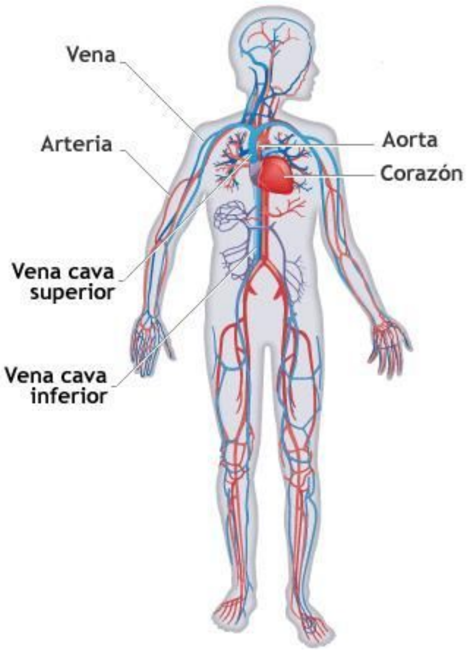


Sangre

Corazón

Arterias

Presión Arterial

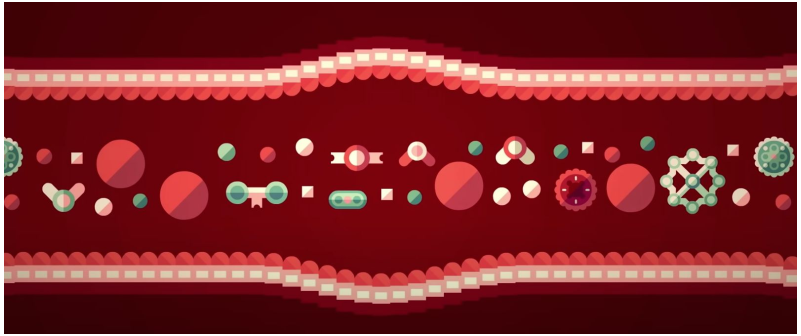

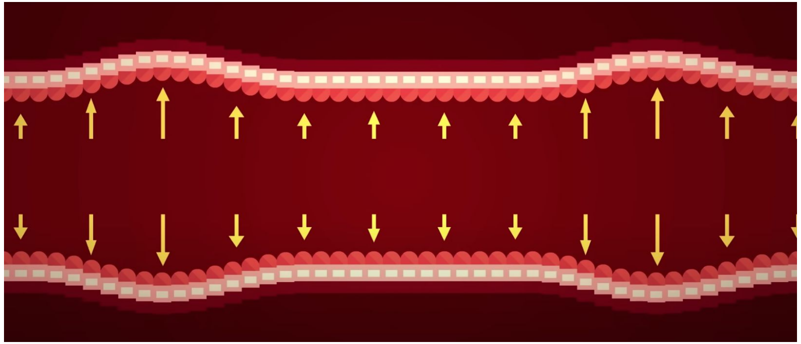

# Tomar la presión arterial

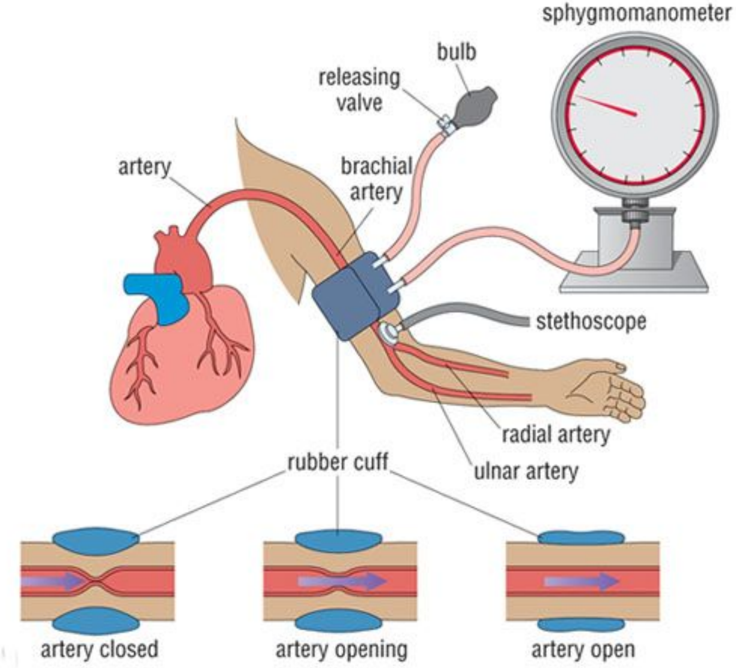


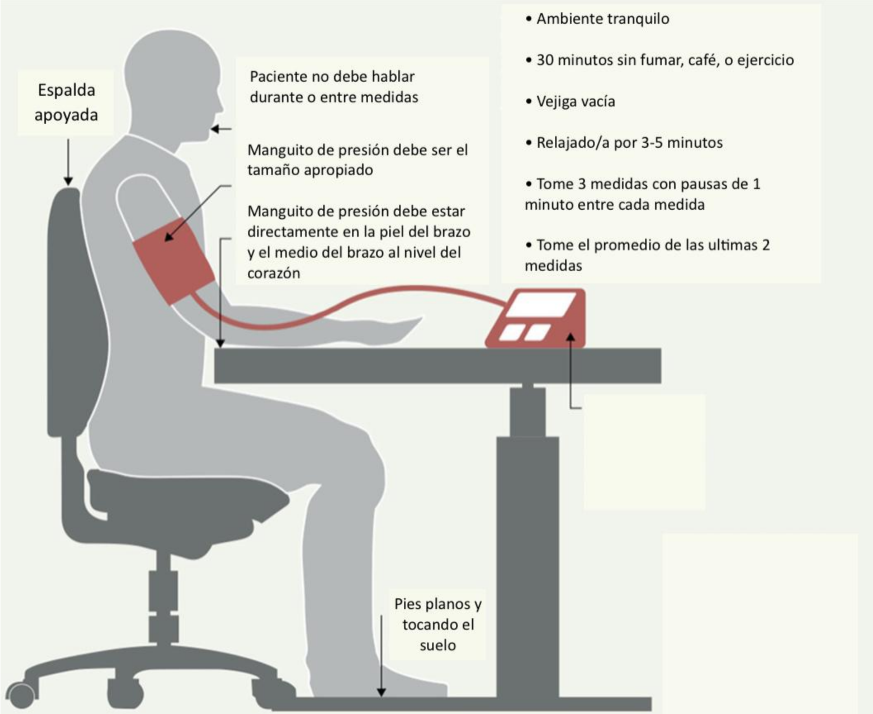

# Sistólica y diastólica

**Sistólica**

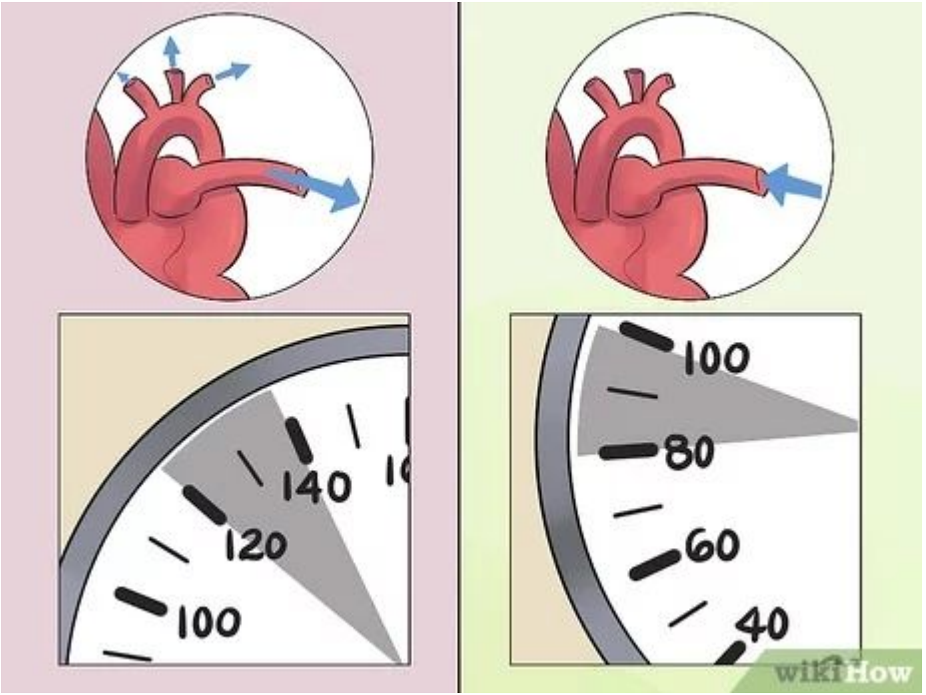


**Diastólica**

# Pregunta

**¿Cuál número representa la presión sistólica?**

**¿Cuál número representa la presión diastólica?**

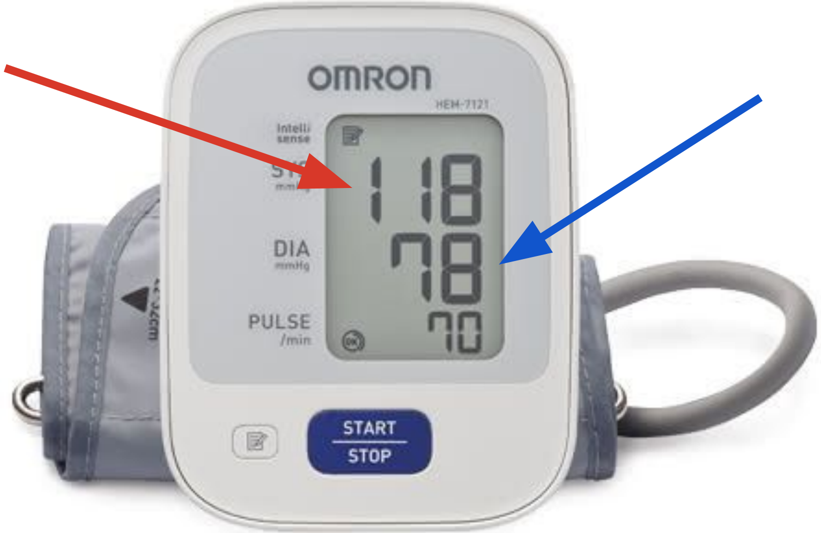

# Respuesta

**Presión sistólica**

**Presión diastólica**

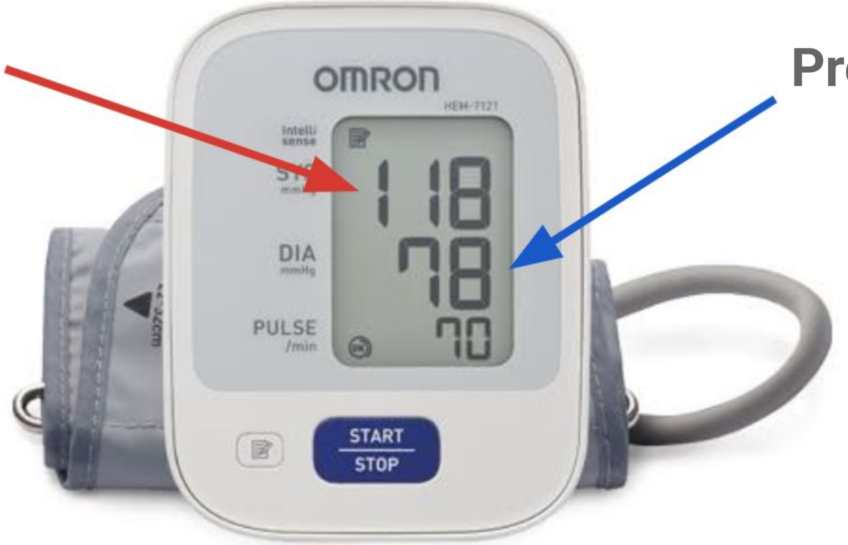

# Hipertensión

# ¿Por qué es peligroso?

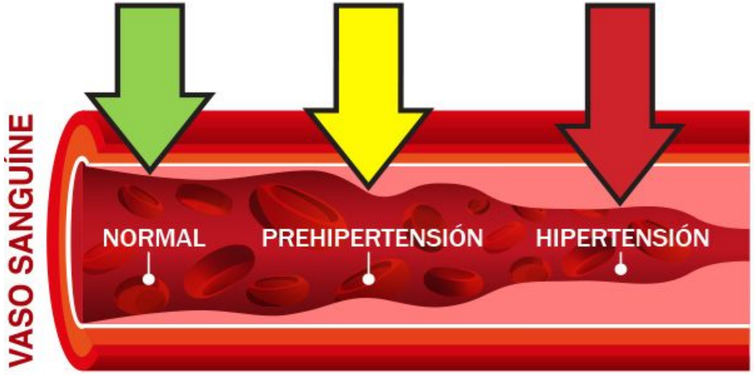


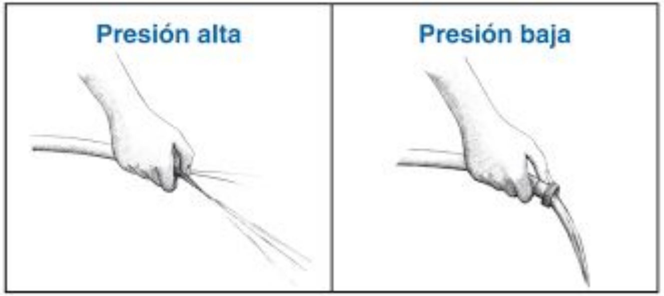


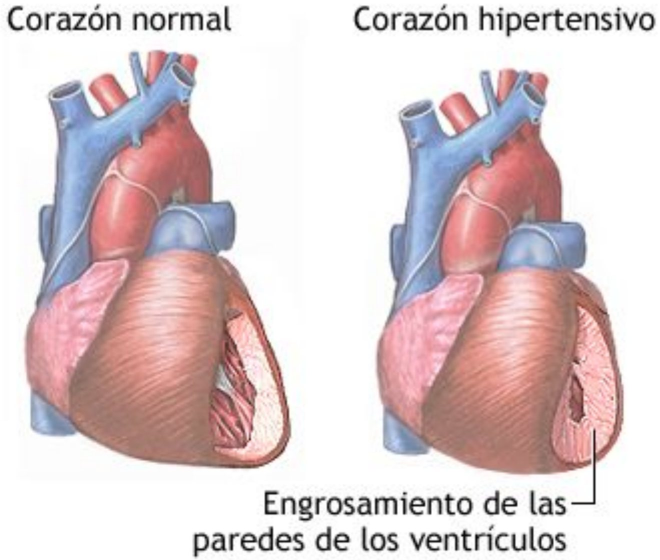

# ¿Por qué es importante?

*Si hay demasiada presión → puede afectar las paredes de las arterias → se pueden dañar y luego bloquear*

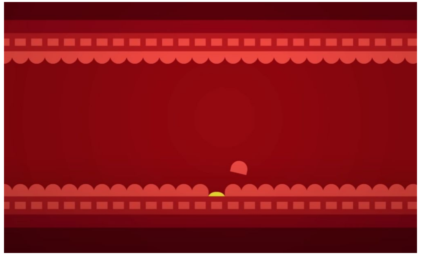

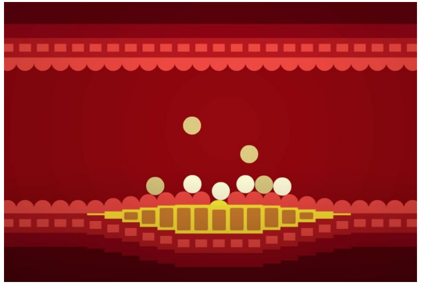

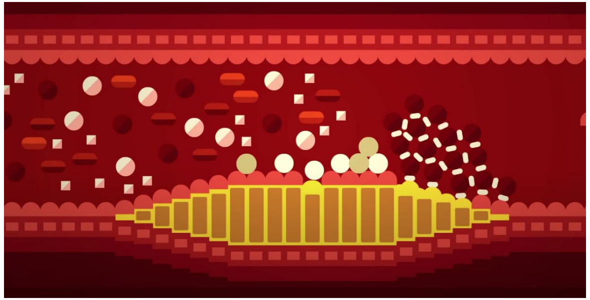

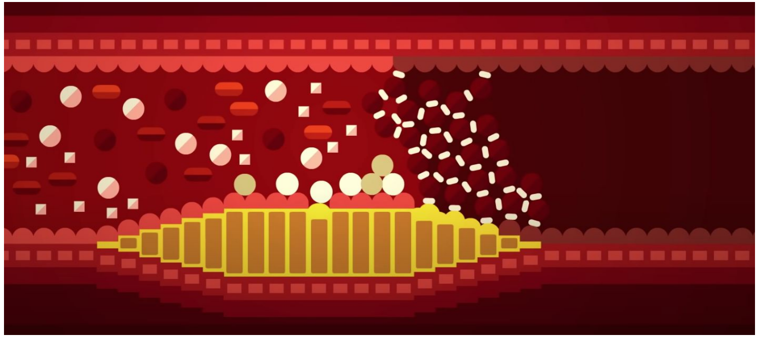

# Diagnosticando hipertensión

Presión de **140/90** mmHg o arriba

En **2** o más ocasiones

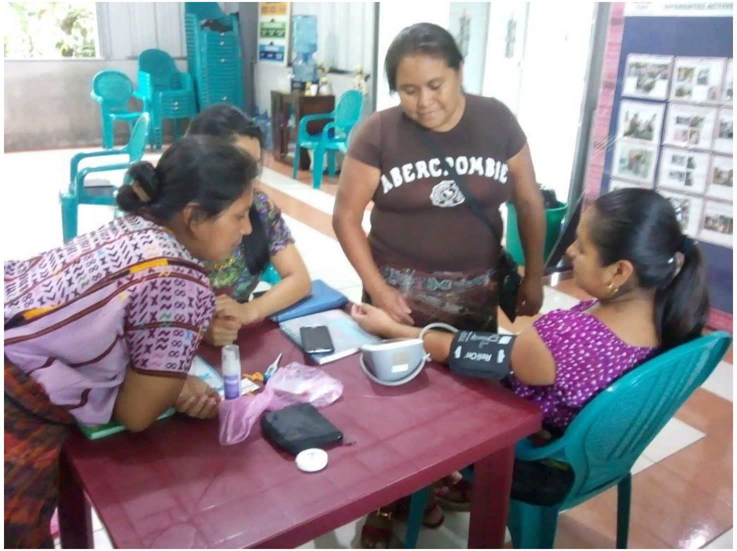

Notas:

- Se puede diagnosticar con solo una visita si esta muy elevada (180/110 o más)
- Pacientes ya diagnosticados y tomando medicamentos

# Niveles de hipertensión

**Normal**

- Debajo de 130/85 mmHg

**Prehipertension**

- 130-139/85-89 mmHg

**Hipertensión**

- 140/90 mmHg o arriba

**Emergencia Hipertensiva**

- 180/110 mmHg o arriba **con síntomas**

| Categoría | PA Sistólica | PA diastólica |
|---|---|---|
| Optima | <120 | <80 |
| Normal | 120-129 | 80-84 |
| Prehipertension | 130-139 | 85-89 |
| HTA grado 1 leve | 140-159 | 90-99 |
| HTA grado 2 moderada | 160-179 | 100-109 |
| HTA grado 3 severa | ≥180 | ≥110 |

# Síntomas de hipertensión

- 
- Normalmente no tiene síntomas
- Cuando tienen síntomas es porque tienen emergencia hipertensiva o tienen complicaciones de hipertensión

# Causas de hipertensión

- Historia familial
- Edad
- Dieta con alto sodio
- Fumar
- Inactividad física
- Estrés constante
- Obesidad y sobrepeso
- Embarazo
- Medicamentos
- Alcohol en exceso

# Pregunta

Toma la presión de su paciente y sale en 145/95 pero su paciente dice que se siente normal.

¿Qué le debe decir sobre los síntomas de la hipertensión?

# Respuesta

- La hipertensión usualmente no presenta con síntomas hasta que hay complicaciones, a veces irreversibles
- Eliminando posibles causas y tomando medicamento puede ayudar a prevenir estas complicaciones y mantenerlos saludables

# Complicaciones de la hipertensión

# Derrame cerebral

- **Definición** → se detiene el flujo de sangre a parte del cerebro debido a un coágulo de sangre o una hemorragia

- **Causas** → presión alta, diabetes, enfermedad del corazón, fumar

- **Síntomas** → debilidad de la cara/brazo, dificultad con hablar, confusión, dolor de cabeza severo, mareos, cambios en la vista

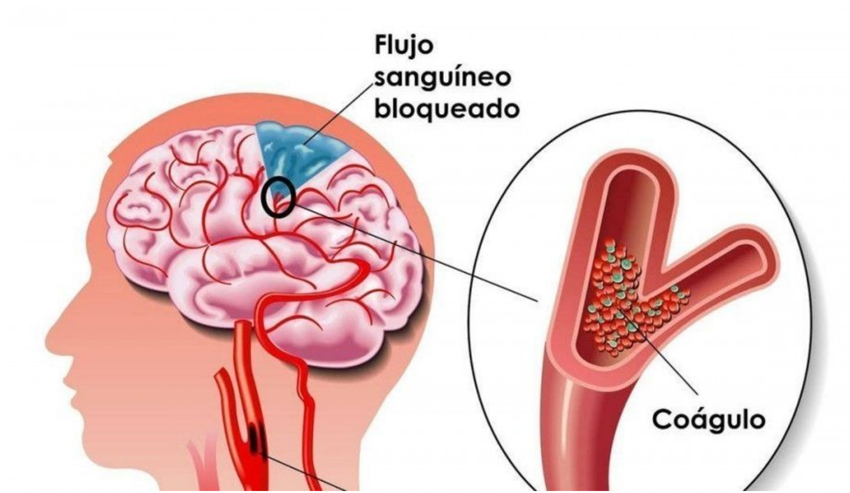

# Enfermedad renal crónica

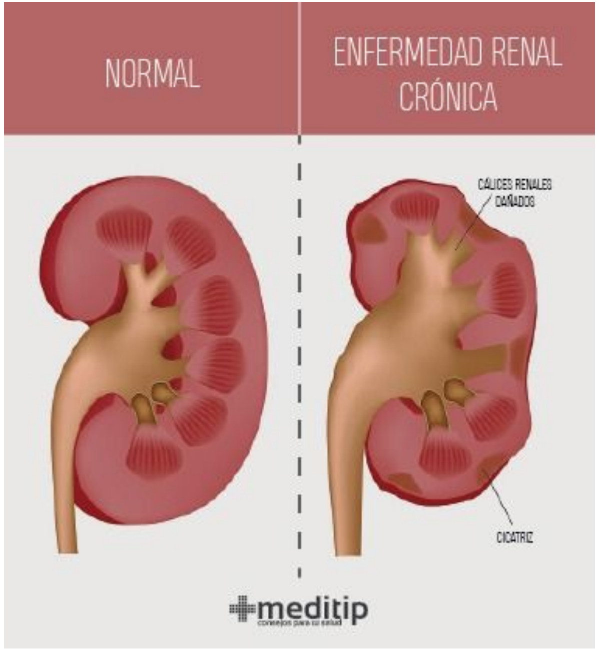


- **Definición** → riñones están dañados y no pueden filtrar la sangre bien
- **Causas** → presión alta, diabetes
- **Síntomas** → típicamente nada específico (hinchazón en las piernas, cansancio, debilidad, orina menos que normal)

# Ataque del corazón

- **Definición** → el flujo de sangre al corazón se bloquea y parte del corazón se empieza a morir

- **Causas** → enfermedad arterial coronaria

- **Síntomas** → dolor de pecho, dificultad al respirar

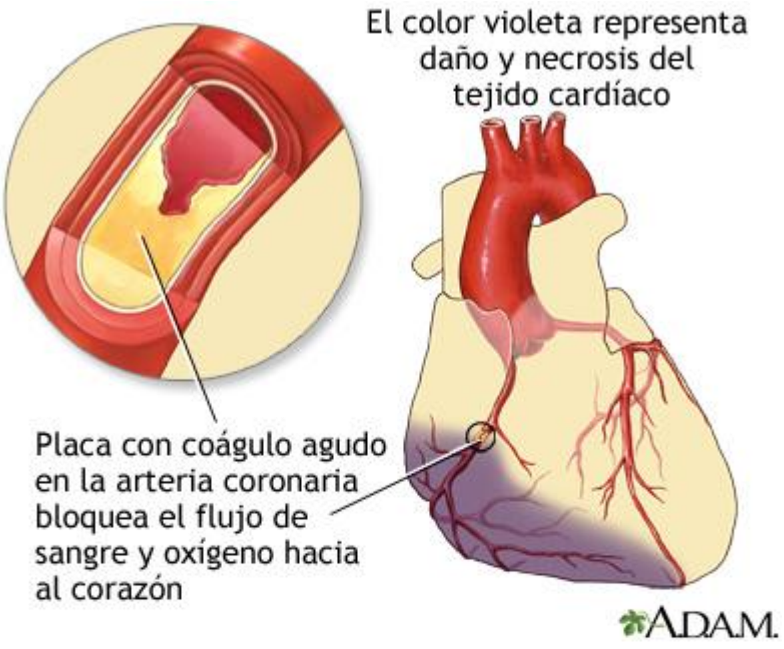

# Fallo cardiaco

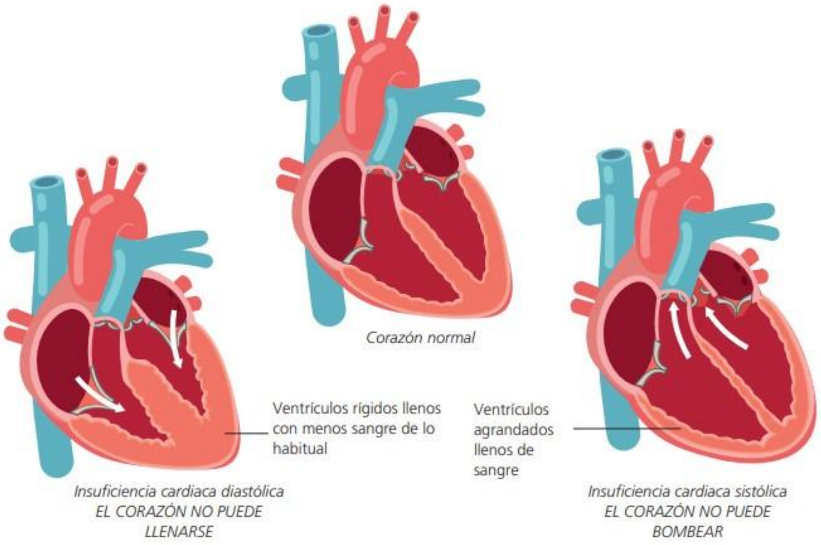


- **Definición** → cuando el corazón no puede bombear sangre al cuerpo correctamente
- **Causas** → presión alta, diabetes, enfermedad arterial coronaria
- **Síntomas** → hinchazón en los tobillos/pies o abdomen, aumento repentino de peso, falta de aire con esfuerzo o acostarse

# Su corazon

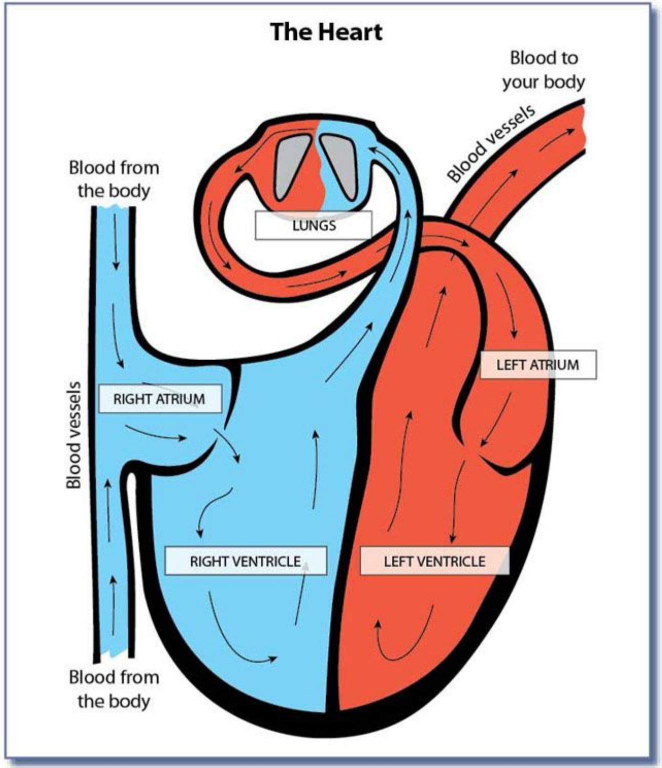

The Heart
Blood to
your body
Blood vessels
Blood from
the body
LUNGS
LEFT ATRIUM
RIGHT ATRIUM
Blood vessels
RIGHT VENTRICLE
LEFT VENTRICLE
Blood from
the body

# Examen fisico

Edema (Hinchazón) con fóvea

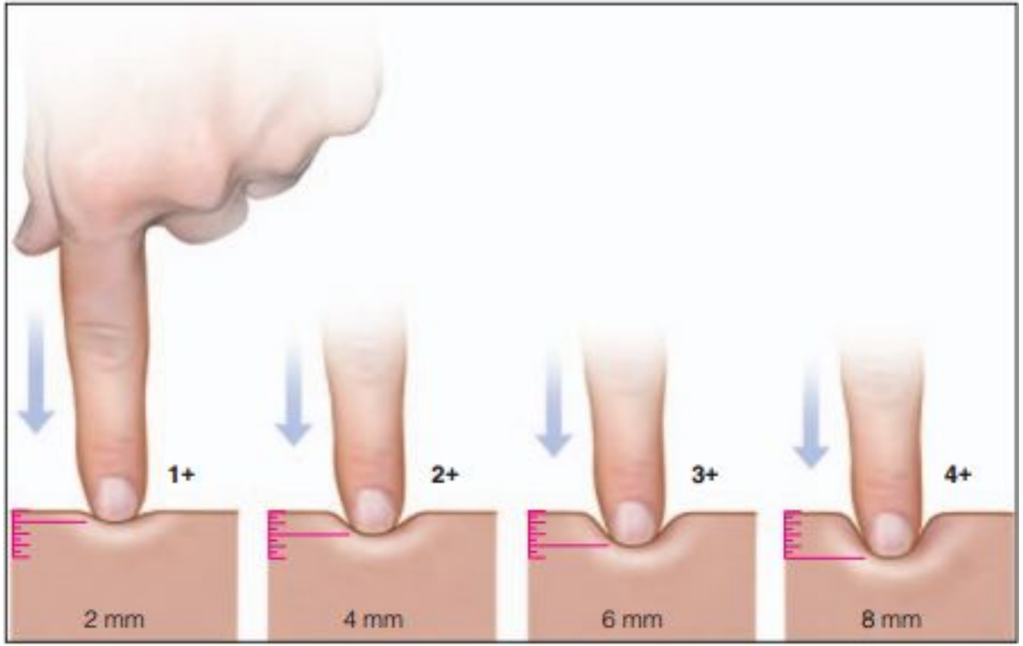


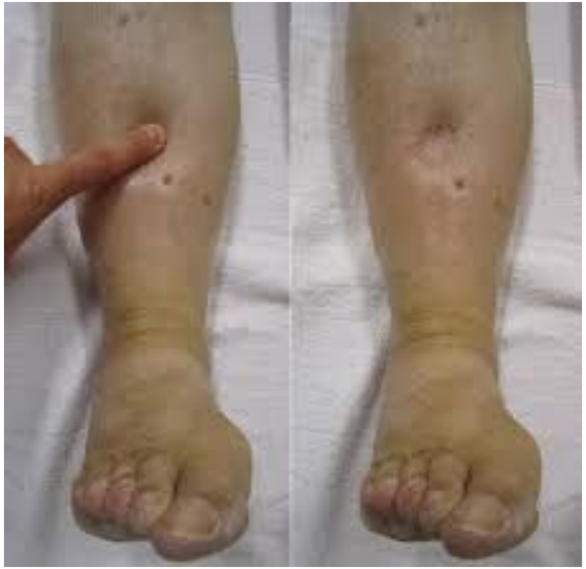

# Oximetro de pulso

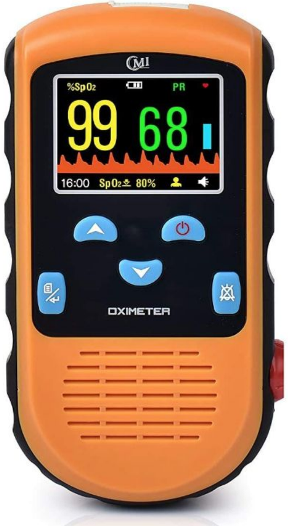


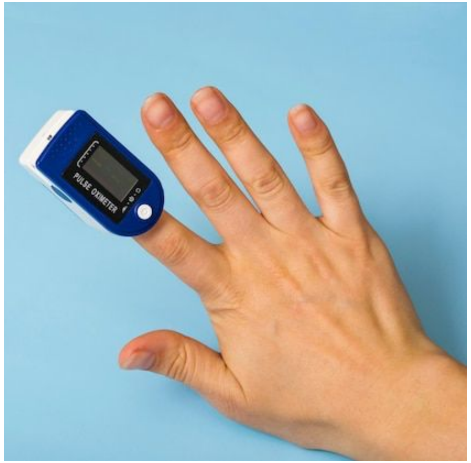


- Muestra la cantidad de oxígeno en la sangre
- Sin oxígeno, el cuerpo no funciona!
- Normal: Mas de 90%
- Si su paciente tiene bajo oxígeno y/o síntomas, consulte un médico

# Emergencia hipertensiva

- Presión sistólica ≥180 mmHg
- Presión diastólica ≥110 mmHg
- **Y tiene síntomas**

- **Síntomas**
  - Dificultad respirando
  - Dolor del pecho
  - Convulsiones
  - Nauseas/vomito
  - Dolor de cabeza severo con visión borrosa
  - Síntomas de un derrame (debilidad en un lado del cuerpo, dificultad con hablar)
  - Letargo

- **Tratamiento**
  - Medicamentos que bajan la presión arterial rápido, pero en manera controlada

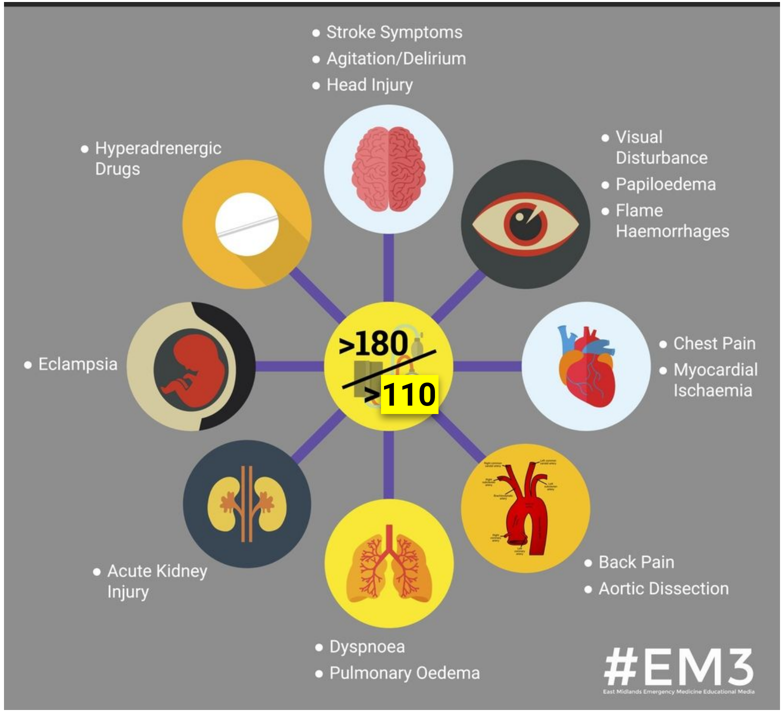

# Hipotensión (presión arterial baja)

- Presión sistólica menos que 90
- Sintomas
  - **Puede ser que no tiene síntomas**
  - Mareos
  - Desmayo
  - Cansancio extremo
  - Nausea severa
  - Confusion
- Causas
  - Medicamentos
  - Deshidratación
  - Infeccion
- Tratamiento
  - Tomar mas agua
  - Posiblemente bajar la dosis de su medicamento
  - Acostarse con piernas levantadas y hablar con médico si tiene síntomas

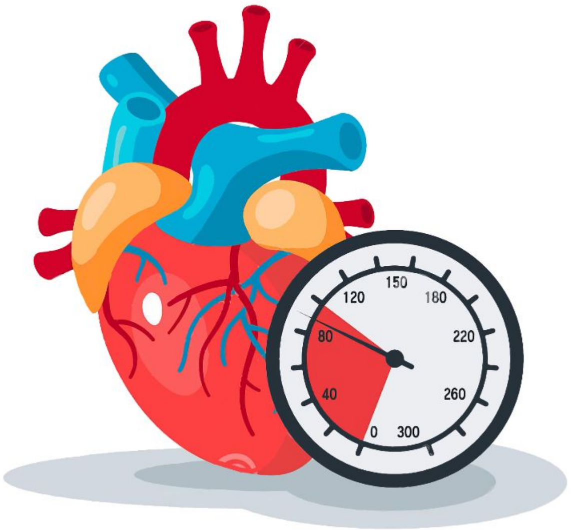

# Hipotensión Ortostática

- Cuando la presión baja demasiado al pararse
  - Presión sistólica baja 20 puntos o más
  - Presión diastólica baja 10 puntos o más
- Síntomas igual a hipotensión pero al pararse
- Diagnosticar
  - 1. Asegúrese que su paciente esté sentado por 5 minutos
  - 2. Tome la presión y pulso del paciente sentado
  - 3. Haga que su paciente se pare
  - 4. Después de 1 minuto parado, tome la presion y pulso otra vez
  - 5. Después de 3 minutos parados, tome la presion y pulso de nuevo
  - 6. Compare la presión antes y después de pararse

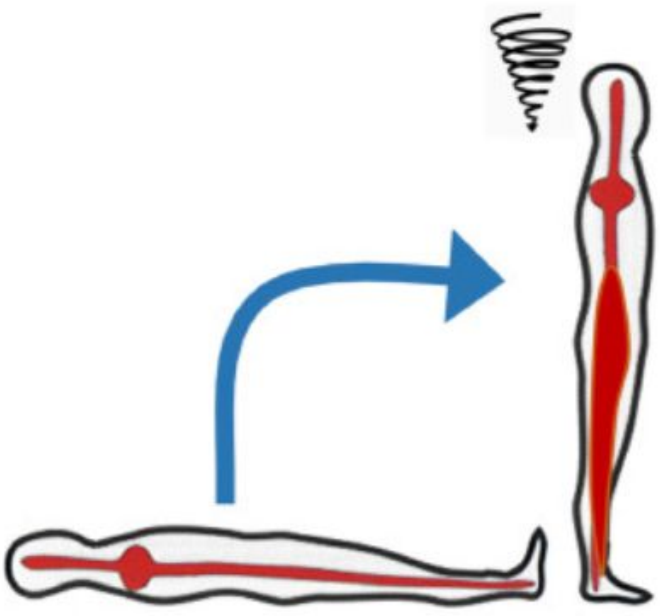

# Bradicardia y taquicardia

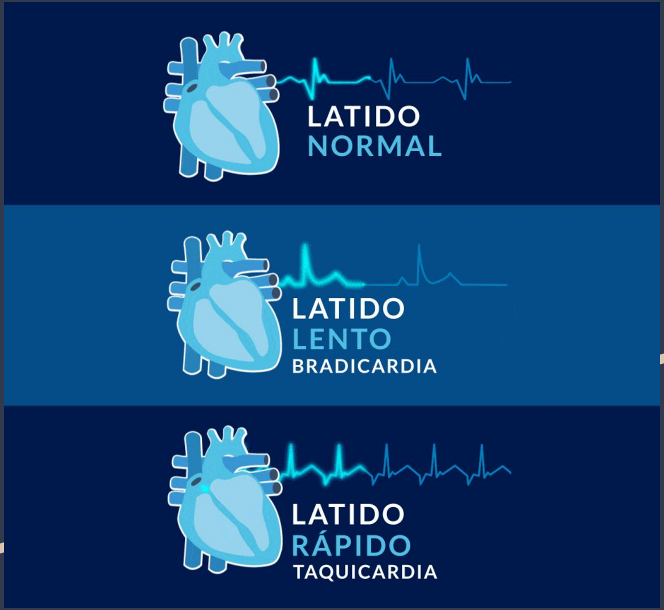


- Pulso normal: 60 - 100
- Hipertensión puede provocar problemas con el ritmo
- Bradicardia severa: pulso 40 o menos
- Taquicardia severa: pulso 120 o mas
- Síntomas: Confusión, dolor de pecho, dificultad para respirar, mareos
- ¡Puede ser emergencia!

# Pregunta

**Su paciente tiene la presión muy elevada pero dice que se siente normal y prefiere no tomar medicamentos.**

**Cuáles son algunos de los riesgos de no tratar su hipertensión que le puede mencionar a su paciente?**

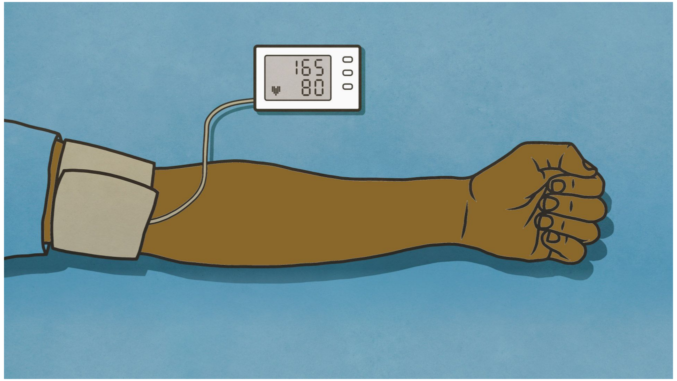

# Respuesta

- Derrame cerebral
- Enfermedad renal
- Ataque al corazón
- Fallo cardiaco
- Trastornos del ritmo cardiaco

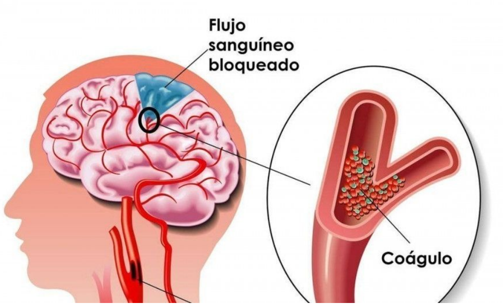


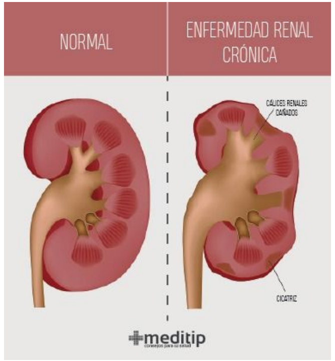


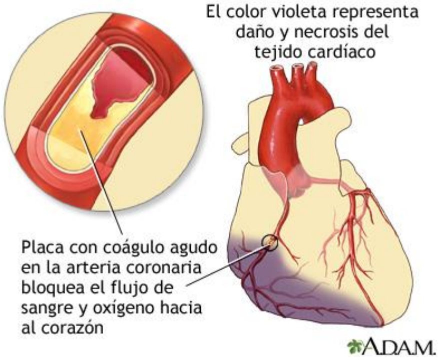

# Identificando un derrame cerebral

# Derrame Cerebral

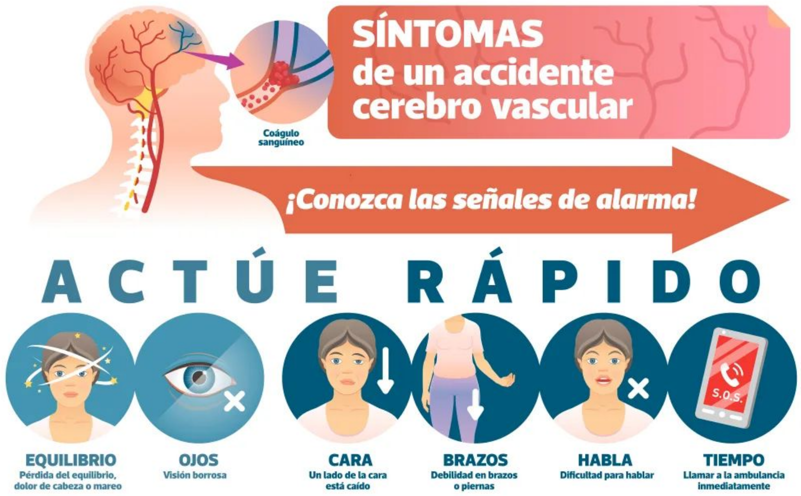


- Cuando el flujo de sangre al cerebro para debido a un coágulo de sangre o hemorragia
- Es una **EMERGENCIA** y debe saber cómo identificarla
- Las siguientes son las señales de alarma para un derrame cerebral..

# Rostro Caído

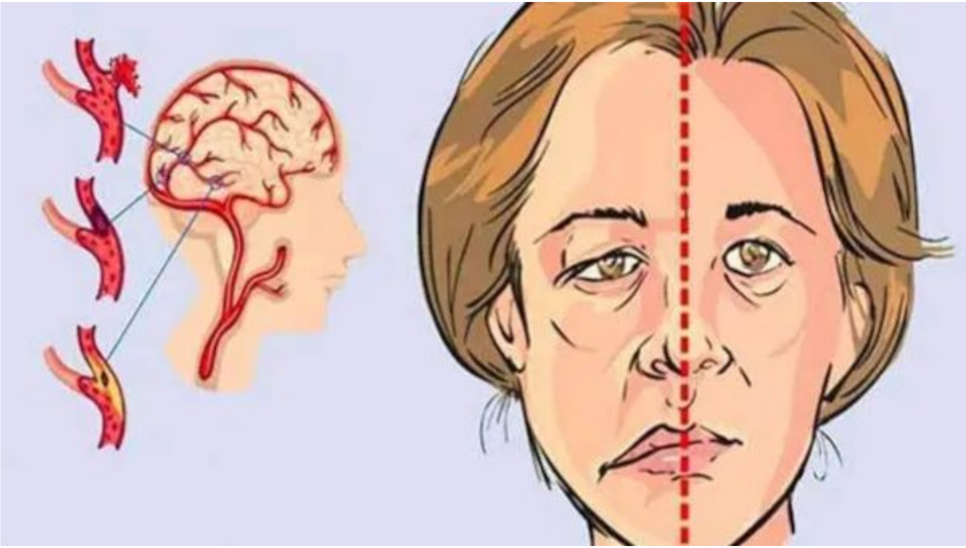

- Cuando alguien está sufriendo de un derrame cerebral, un lado de su cara estará caída

- **Exámen**:
    - Pídale al paciente que sonría
    - Si un lado de su sonrisa está caída, esto pude ser señal de un derrame

# Pérdida de Fuerza

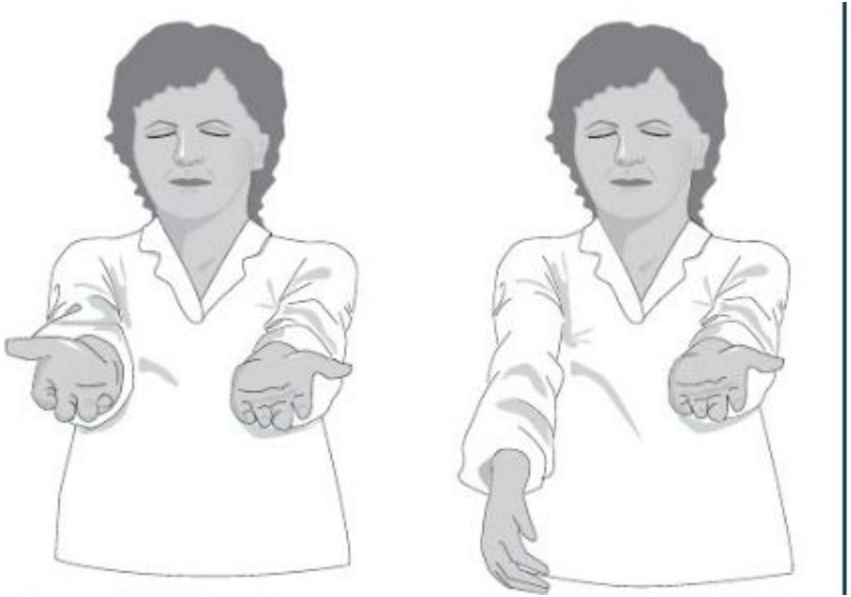

- Cuando alguien está sufriendo de un derrame cerebral, los brazos y piernas están débiles en un lado del cuerpo

- **Exámen:**
    - Pídale al paciente que cierre los ojos y extiende sus dos brazos con las palmas arriba por 10 segundos
    - Si un lado no puede subir normalmente o se caiga, esto puede ser una señal de un derrame

# Dificultad para Hablar

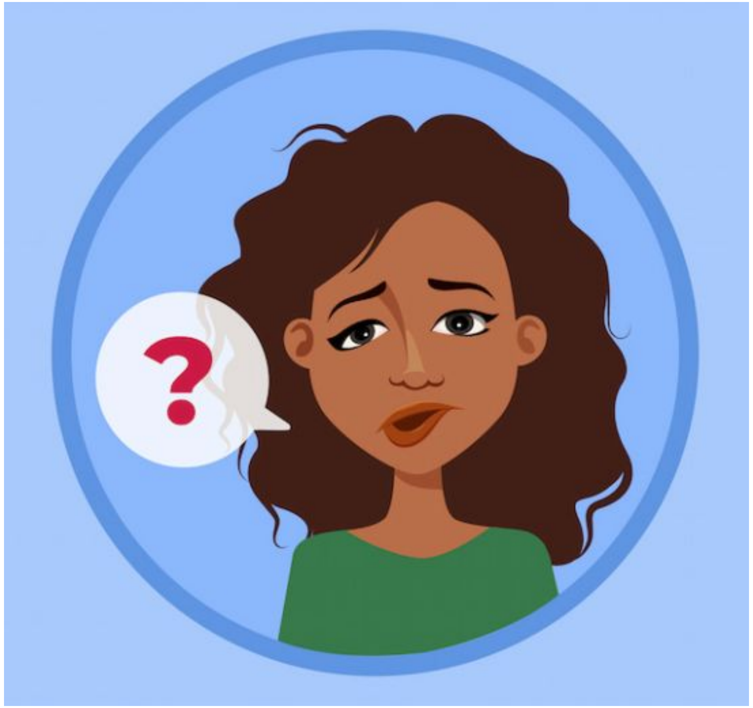


- Cuando alguien está sufriendo de un derrame cerebral, puede tener dificultades al hablar o entender lo que le están diciendo

- **Exámen:**
  - Pídale al paciente que repita unas frases/palabras - “mitad y mitad”, “gracias”, “futbolista”
  - Si el paciente arrastra las palabras, dice palabras incorrectas o no puede hablar, esto puede ser señal de un derrame

# ¡Obtenga Ayuda!

- Si el paciente demuestra **cualquiera** de estas señales, ¡obtenga ayuda inmediatamente!

- Un derrame cerebral es una **EMERGENCIA** y el paciente necesita tratamiento

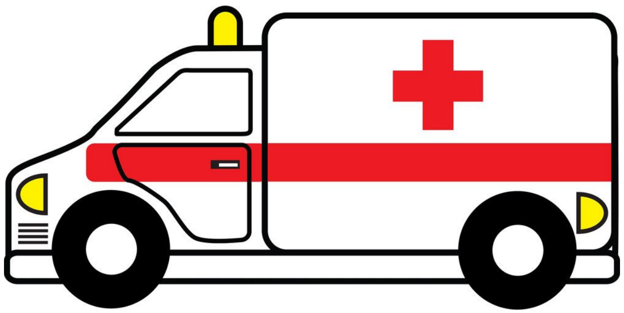

# Pregunta

**Su paciente viene a la clínica y <u>su rostro no se ve normal</u> cuando trata de sonreír.**

**¿Qué debe hacer usted para evaluar si su paciente está sufriendo de un derrame?**

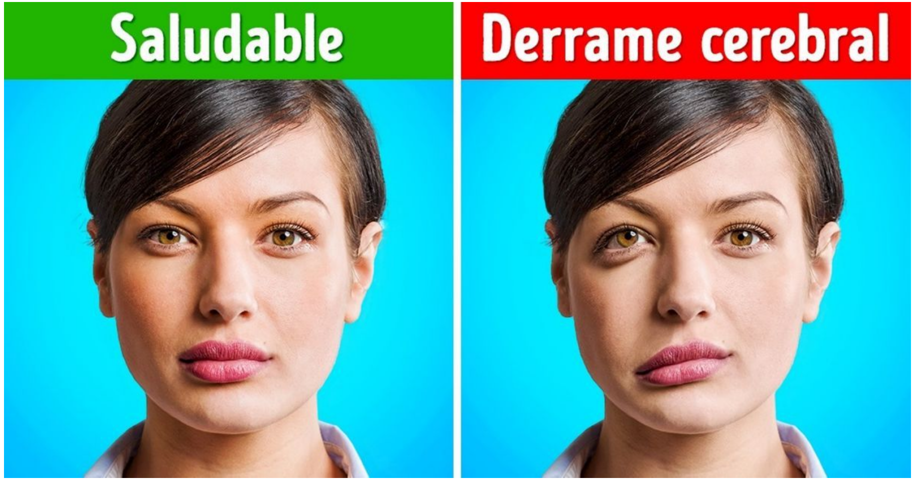

# Respuesta

1. Pedirle al paciente que **levante los dos brazos**

2. Pedirle al paciente que **repita una frase simple**

## ***Ojo***

Si observa cualquiera de estos síntomas, ¡llame al 911 inmediatamente!

# Prevención y tratamiento de hipertensión

# Sodio

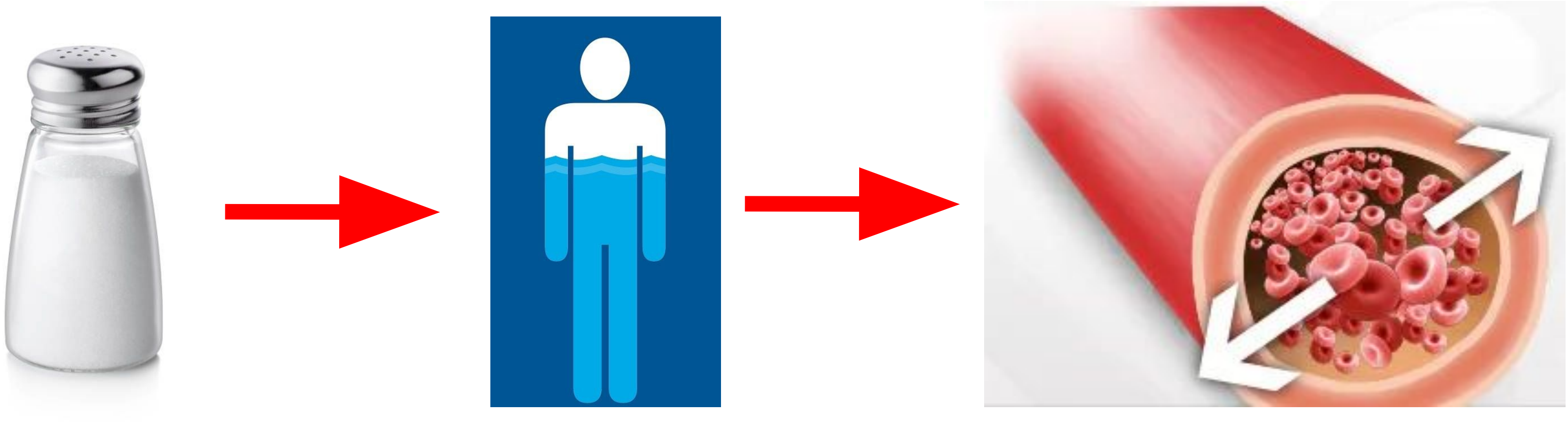

# Para reducir el sodio..

**En vez de:**

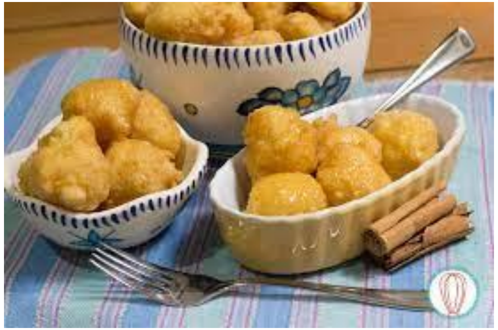

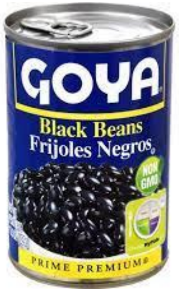


**Escoja:**

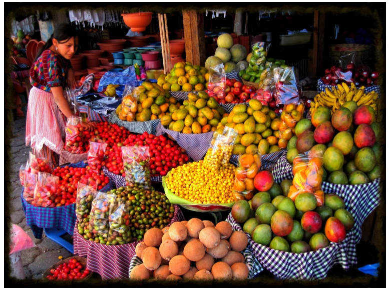

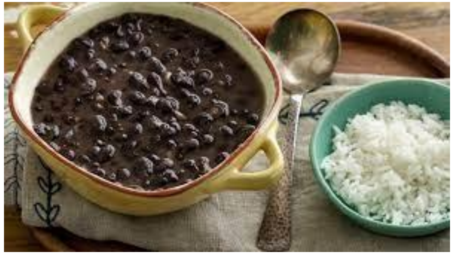

# Para reducir el sodio..

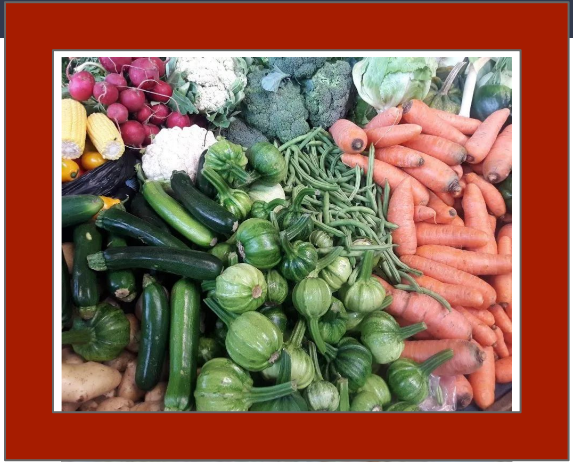

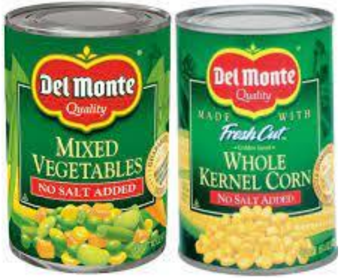



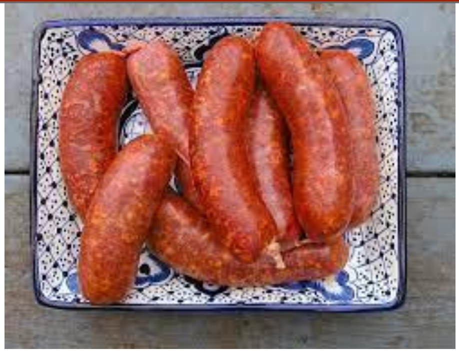

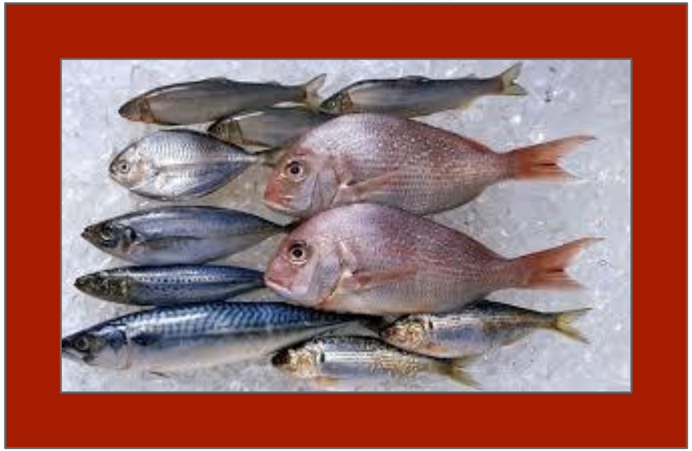

# Dieta Sana - Frutas y Verduras

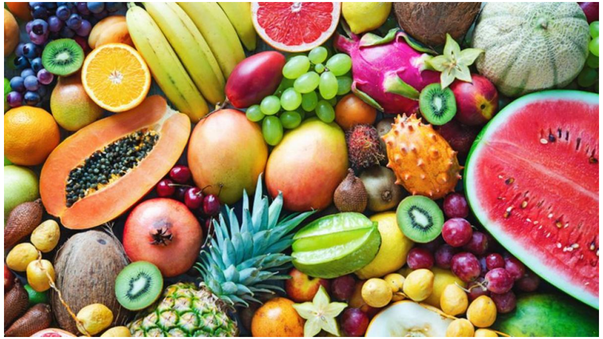

- Los alimentos más importantes

- Tienen muchas **vitaminas** y **fibra** que ayudan a llenarse y a controlar la azúcar y la presión

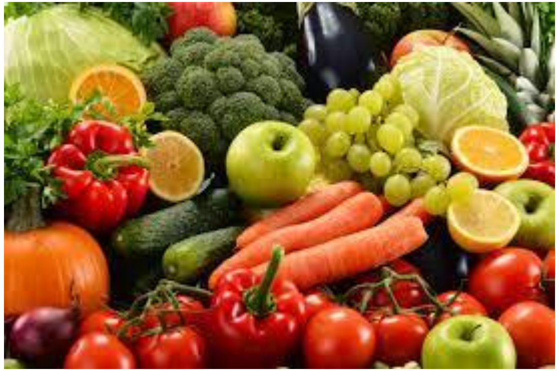

- Deben ser ½ del plato en cada comida

- Fruta entera (no jugo) puede reemplazar a los dulces o postres

# Dieta Sana - Carbohidratos

- Se necesita **1-2 porciones** de carbohidratos saludables por comida

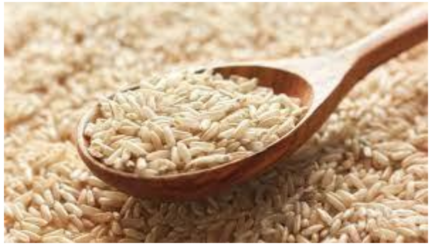

- Ejemplos de una porción:
  - 1 tortilla
  - Pan integral (del tamaño de un puño)
  - ½ taza de avena sin azucar
  - ½ taza de fideos o arroz integral
  - ½ taza de papas

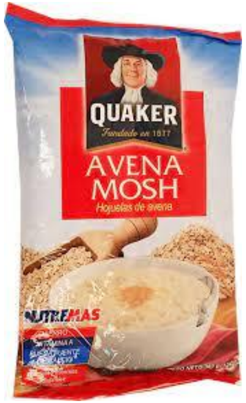


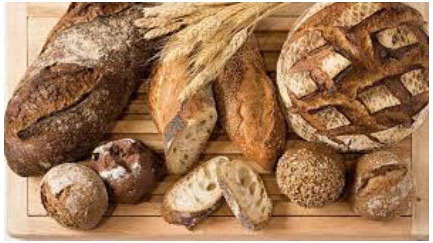

# Dieta Sana - Proteína

- Se necesitan para los músculos y para crear hormonas naturales en el cuerpo

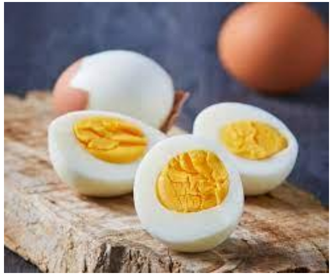

- Se puede usar para energía también

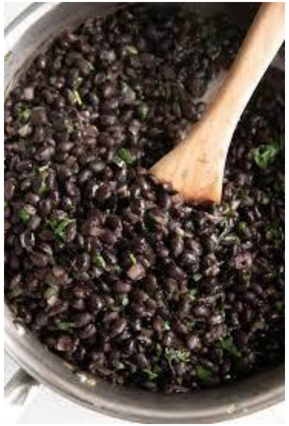

- Un alimento lleno de proteínas saludables en cada comida
    - 1 huevo
    - ½ taza de frijoles
    - pollo sin pellejo (el tamaño de la palma de la mano)
    - Nueces y semillas (¼ taza)

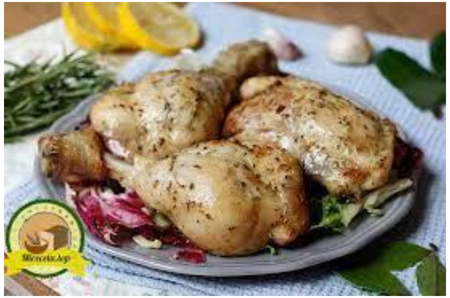

# Mantenerse activo!

- Actividad moderada ~ 30 minutos cada día
  - Subir escaleras, bailar, caminar a paso ligero, jardinería

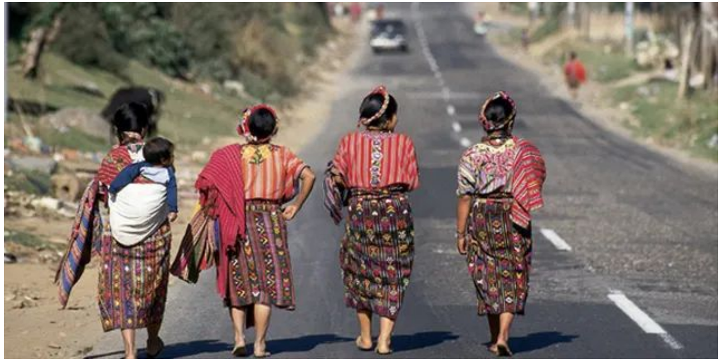

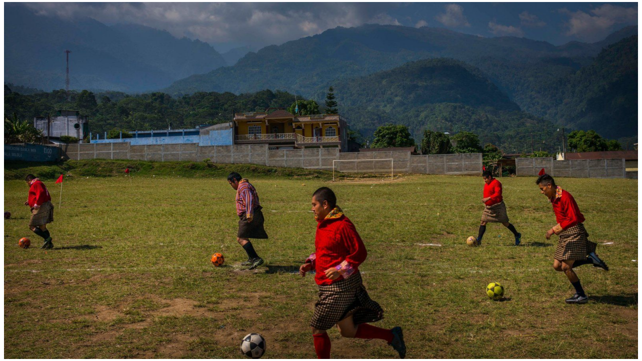

- Actividad vigorosa ~1 hora y media cada semana
  - Correr, montar bicicleta, nadar, practicar deportes

# Fumar y Alcohol

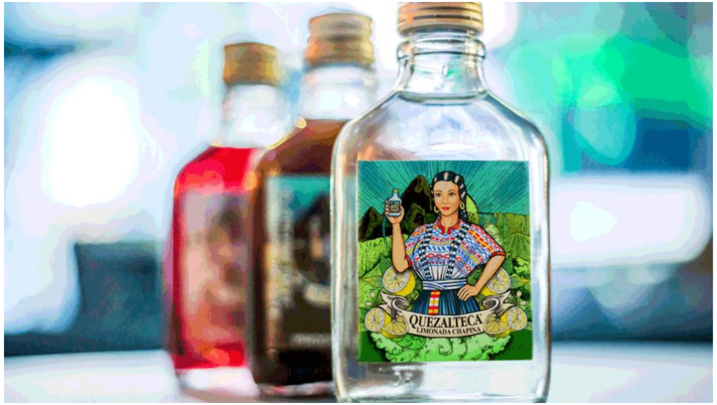


- Alcohol solo en moderación

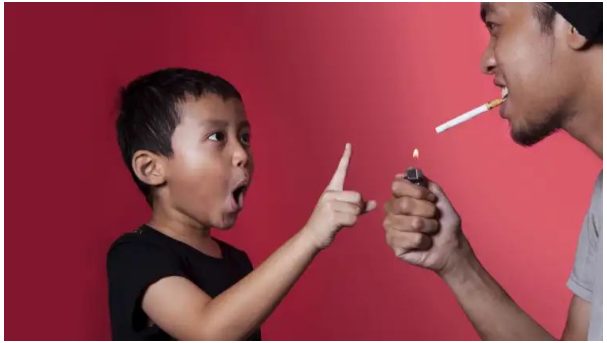

- ¡Un cigarrillo sube la presión por 20 puntos!
- ¡Evitar completamente!

# Medicamentos que provocan hipertensión

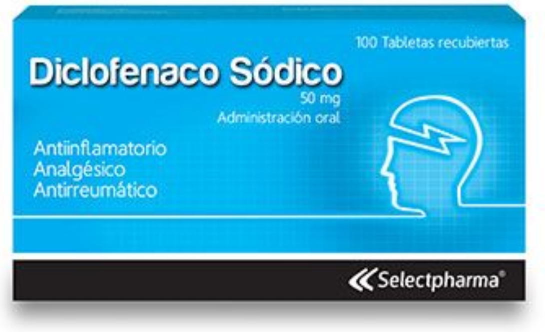


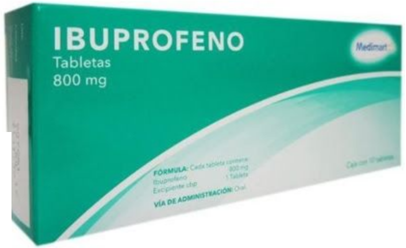


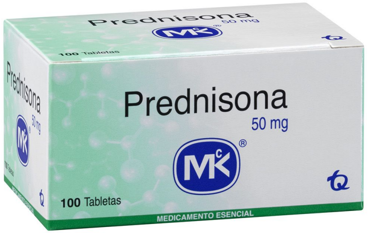


- Antiinflamatorios no esteroideos (AINEs)
  - Diclofenaco, ibuprofeno, naproxeno
  - Acetaminofen no sube presión

- Prednisona y otros esteroides

# Cocinando

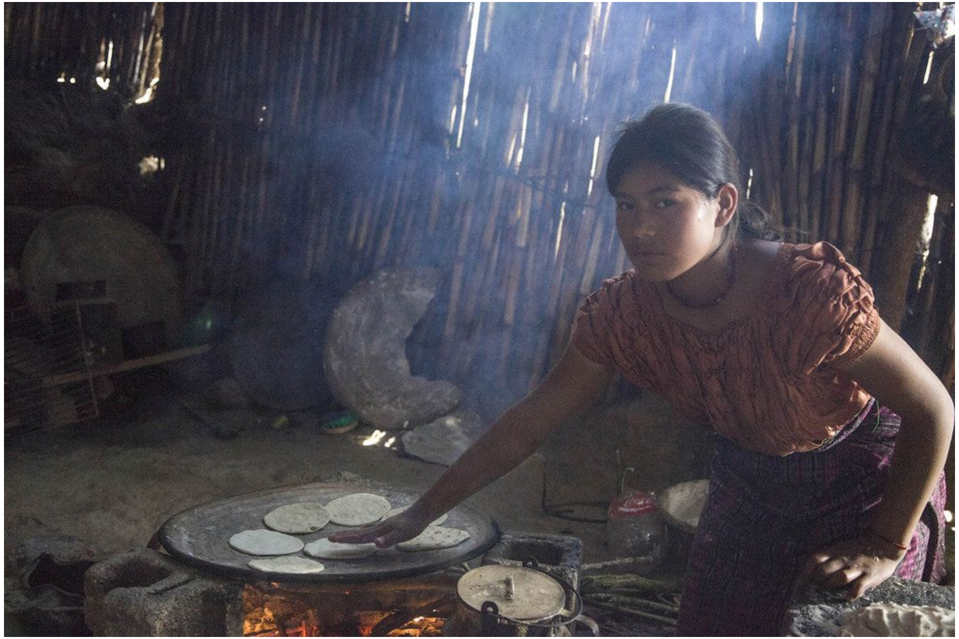

- Humo sube la presión dentro de 24 horas
- Mejor estufa ambiental con chimenea, estufa de gas, o estufa eléctrica

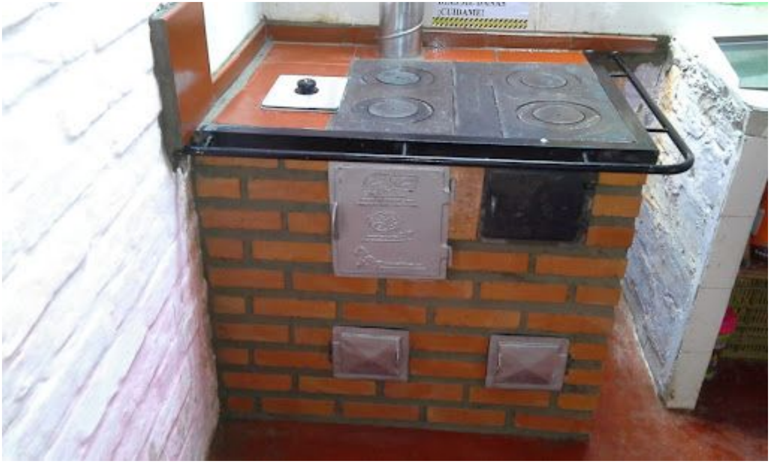

# Pregunta

Su paciente tiene presión elevada y le pregunta sobre su estilo de vida:

- Trabaja largas horas manejando un microbús
- Le encanta comer pollo campero todos los días para el almuerzo
- Fuma 10 cigarrillos todos los días porque dice que le ayuda manejar el estrés de su vida
- Cuando llega a su casa, solo se sienta a ver televisión hasta la hora de dormir

¿Qué sugerencias le daría a su paciente para tratar de bajar su presión?

# Respuesta

- Cigarrillos - Dejar de fumar o empezar por reducir el número que fuma
- Actividad - Empiece por salir a caminar cuando pueda o cuando llegue a casa
- Nutrición - trate de evitar grandes cantidades de sodio todos los días y elija frutas o comidas preparadas en casa

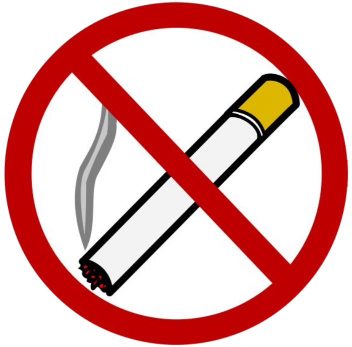

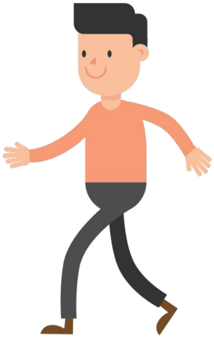

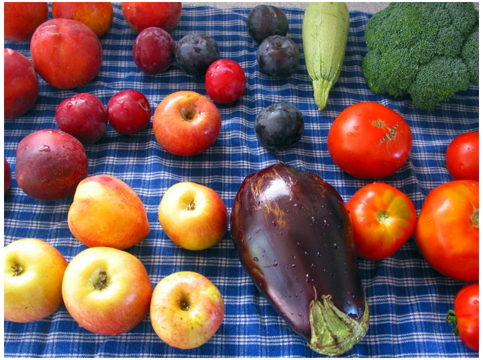

# Introducción a la Iniciativa de Hipertensión: Dia 2

Valerie Aguilar, Estudiante de medicina
Juan Aguirre, Estudiante de medicina
Sean Duffy, MD, MPH
Universidad de Wisconsin

# Agenda

Jueves, 7/21

- Básico de hipertensión
- Complicaciones
- Derrame cerebral
- Prevencion

Miercoles, 7/27

- Medicamentos y efectos secundarios
- Laboratorios
- Comorbilidades
- Practicar medidas de presión
- Práctica de aplicación

# Repaso

# Presión arterial

## ¿Qué mide la presión arterial?

- La presión que la **sangre** le pone a las **arterias**

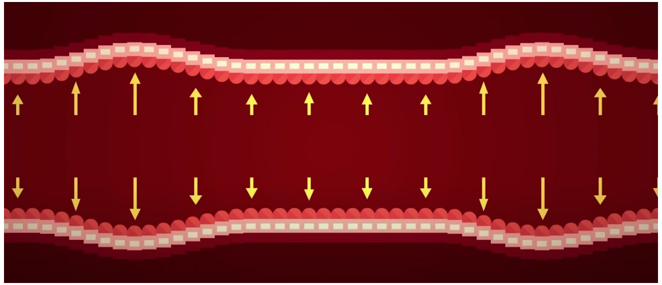

## ¿Es normal, prehipertensión, hipertensión, o emergencia hipertensiva?

- 120/80 — Normal
- 140/95 — Hipertensión
- 130/86 — Prehipertension

| Categoría | PA Sistólica | PA diastólica |
|---|---|---|
| Optima | <120 | <80 |
| Normal | 120-129 | 80-84 |
| Prehipertension | 130-139 | 85-89 |
| HTA grado 1 leve | 140-159 | 90-99 |
| HTA grado 2 moderada | 160-179 | 100-109 |
| HTA grado 3 severa | ≥180 | ≥110 |

# Prevención de la hipertensión

- Caminar

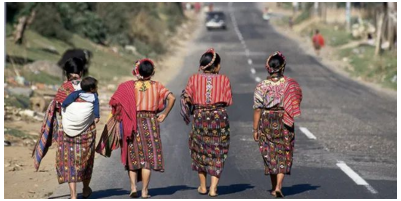

Bueno ✓

- Comer frijoles enlatados

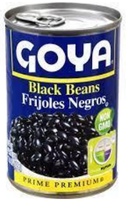


Evitar ✘

- Comer frutas

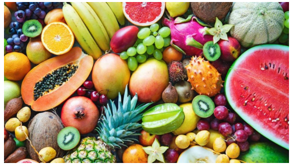

Bueno ✓

- Cigarrillos o cigarros

Evitar ✘

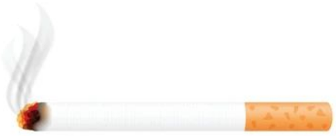

- Cocinar con estufa de gas (sin humo)

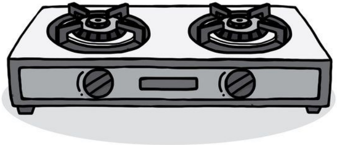

Bueno ✓

- Quetzalteca

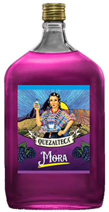

Evitar ✘

# Medicamentos del protocolo para el programa de hipertensión

# Amlodipina

- **Cómo funciona:** Relaja y dilata las arterias
- **Dosis:** 2.5 a 5 mg → puede subir a 10 mg
- **Efectos secundarios:** ***hinchazón de los tobillos/pies***, mareos al pararse, sonrojo, náusea/dolor abdominal, hinchazón de las encías

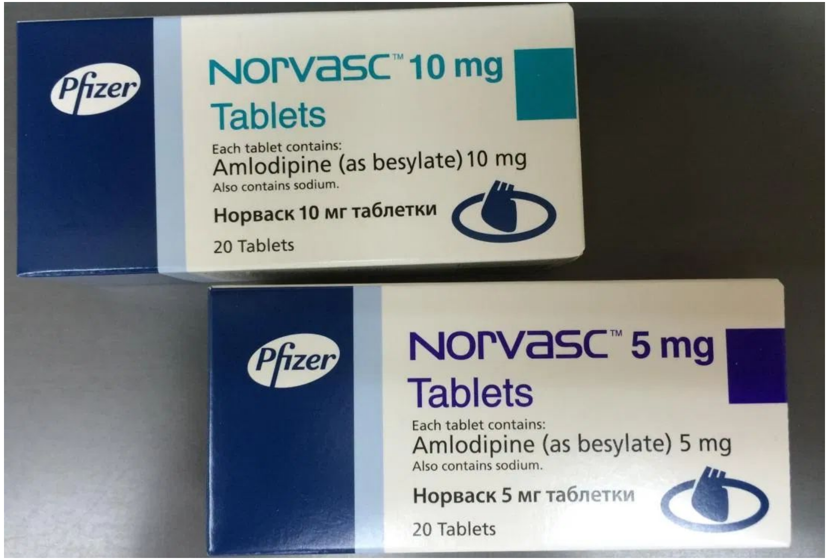

# Hinchazón de los pies

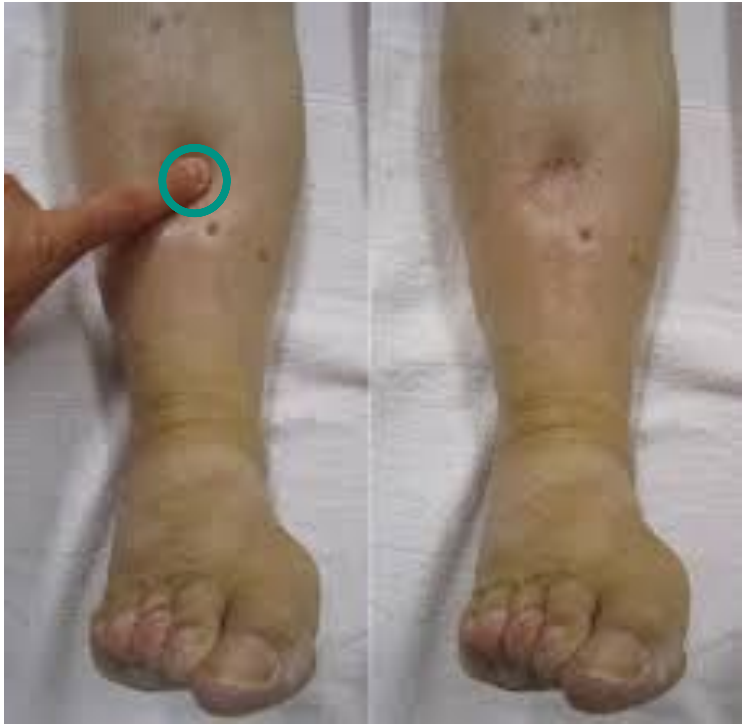

# Hinchazón de las encías por amlodipina

No muy común pero puede
pasar con uso de largo plazo

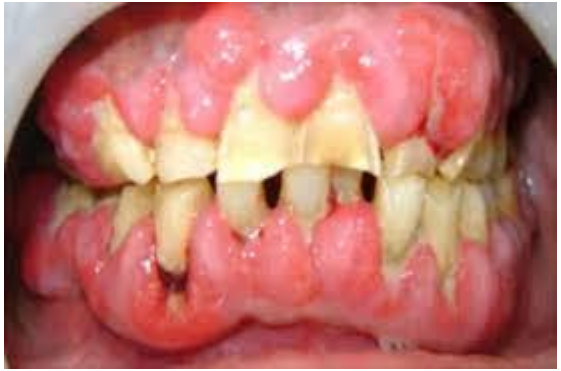

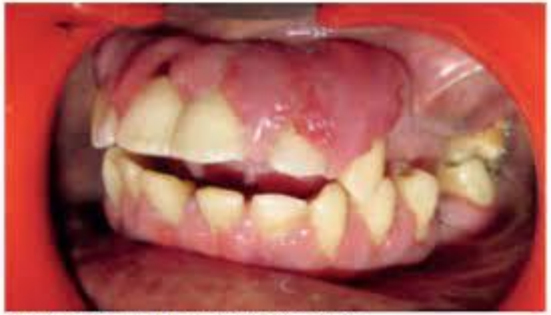

Figure 3. Right lateral view at first visit

# Losartán

- **Cómo funciona:** bloquea una proteína que causa que las arterias se encojan
- **Dosis:** 25 a 50 mg → puede subir a 100 mg
- **Efectos secundarios:** mareos al pararse, potasio alto, problemas con riñones, tos*

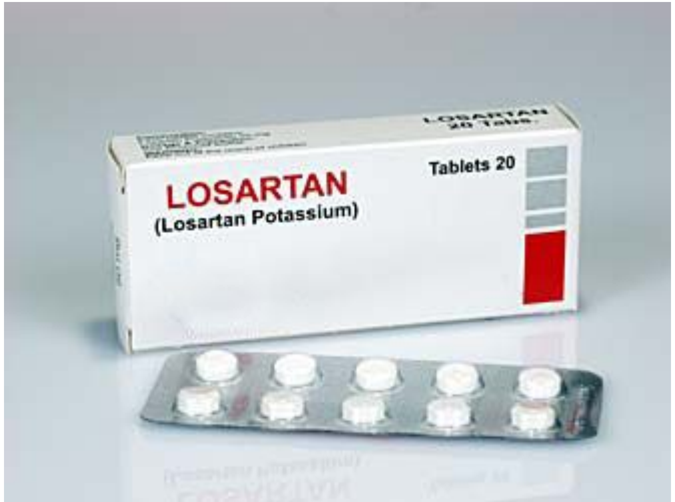


*Mucho menos común que con enalapril

# Enalapril

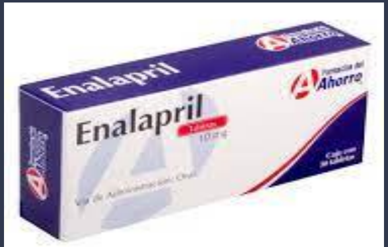


"eNALApril (la perrita de Juan) tiene **tos**"

- **Cómo funciona:** bloquea una proteína en el cuerpo, causando que haya más espacio en las arterias
- **Dosis:** 5 mg al día → puede subir a 40 mg al día
- **Efectos secundarios:** ***tos***, mareos al pararse, potasio alto, problemas con riñones, hinchazón de cara labios, lengua o garganta (raro)

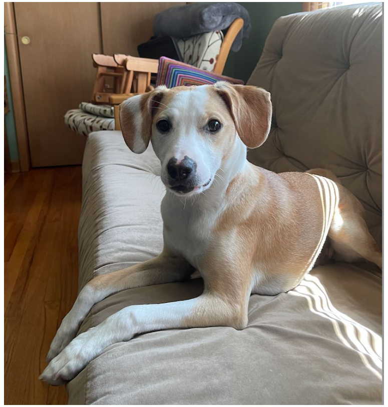

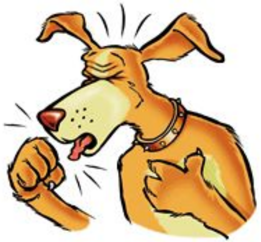

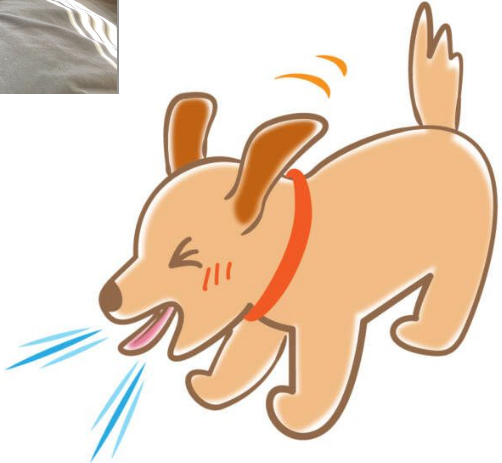

# Hinchazón de cara, labios, lengua o garganta por enalapril (angioedema)

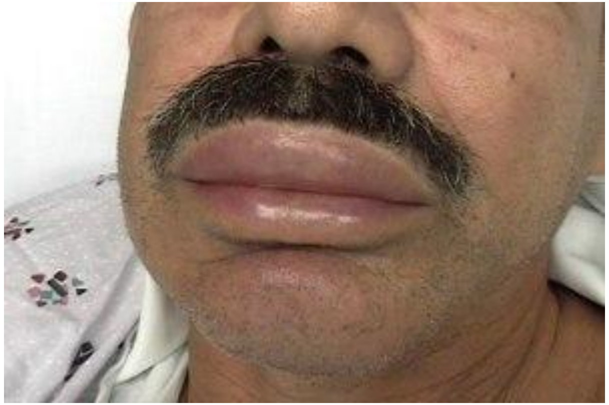
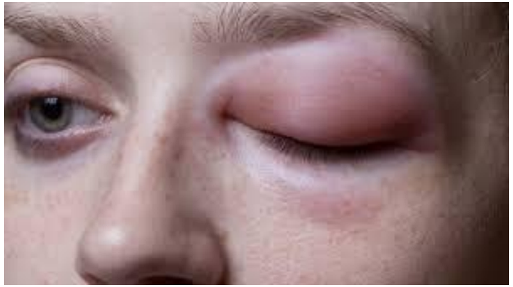
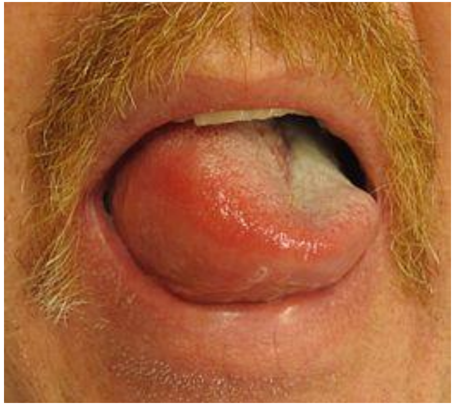

*Si ve hinchazón de la cara o boca y/o dificultad para respirar, puede ser emergencia médica!

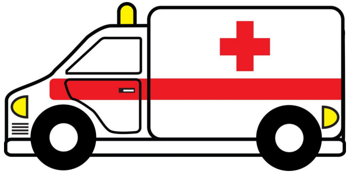

# Atorvastatina (estatina)

“El **tat**uaje que tengo en el **músculo** me **duele**”

- **Cómo funciona:** causa que el hígado deje de hacer colesterol y así ayuda a bajar el colesterol en el cuerpo y baja el riesgo para un ataque al corazón o derrame cerebral
- **Indicación**: Historia de infarto o derrame o riesgo alto para estas complicaciones
- **Dosis:** 10 - 20 mg al dia → 80 mg al dia
- **Efectos secundarios: dolor muscular**, daño del hígado (raro), aumento de azúcar en la sangre

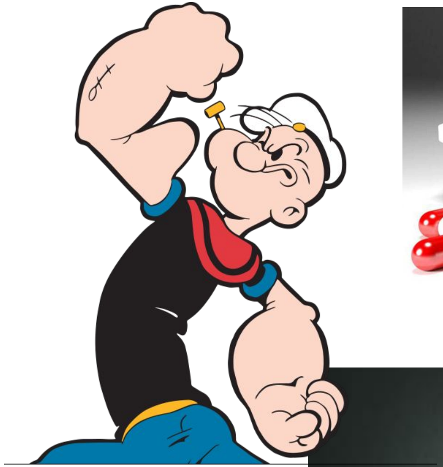

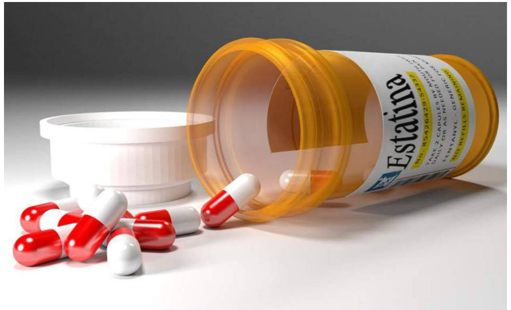


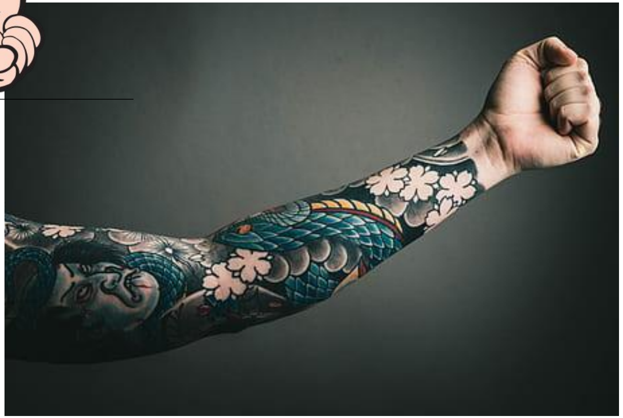

# Medicamentos en las mujeres que pueden embarazarse

- **No se recomienda enalapril o losartán en mujeres que pueden embarazarse** → causan malformaciones congénitas

- **No se recomienda atorvastatina en mujeres embarazadas** → colesterol es importante en el desarrollo del bebe

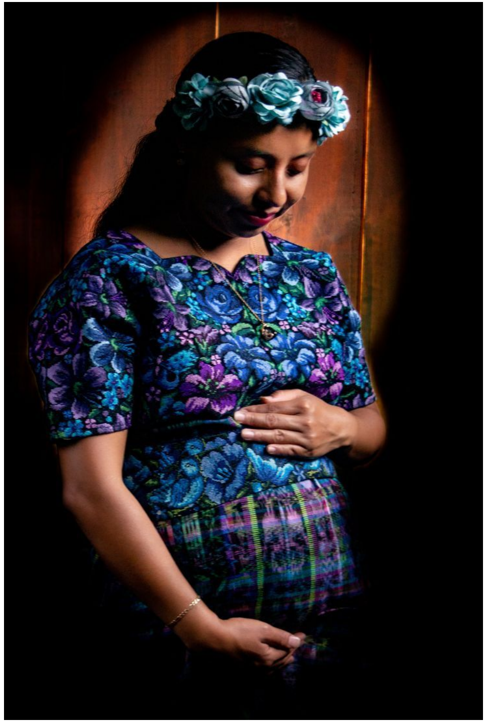

# Aspirina

- **Cómo funciona:**
  - Previene la formación de coágulos en la sangre - así ayuda a prevenir ataques al corazón o derrames cerebrales
  - Ayuda a controlar la hipertensión también al mejorar el flujo de sangre entre las arterias
- **Indicación:** Historia de enfermedad coronaria, infarto o derrame
- **Dosis:** 81 mg al dia (dosis baja)
- **Efectos secundarios:** dolor del estómago, sarpullido, acidez, úlceras en el estómago o el intestino (y sangrado por esto)

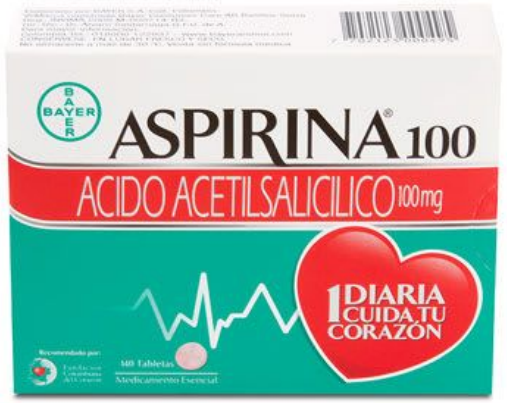

# Pregunta

Su paciente tiene hipertensión y elige darle Enalapril. ¿Cuál es el efecto secundario más común de este medicamento?

# Respuesta

Tos!

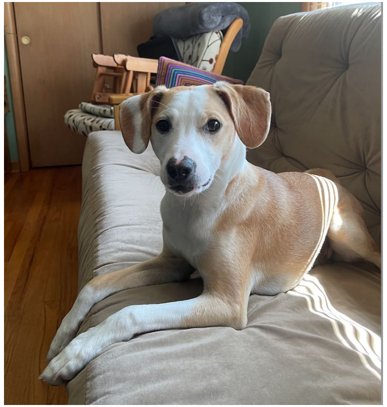

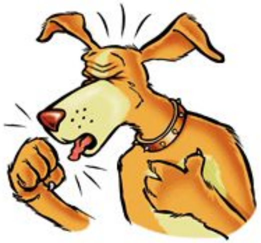

También puede tener mareos al pararse, potasio alto, problemas con riñones, hinchazón de cara labios, lengua o garganta (raro)

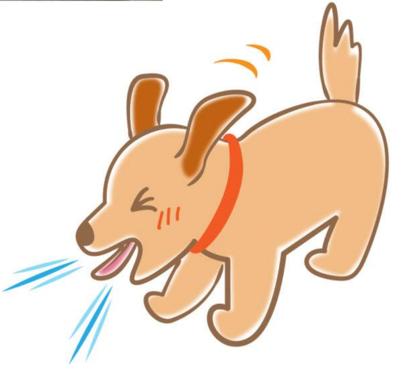

# Medicamentos posibles de sus pacientes

# Hidroclorotiazida

“Oro → el paciente orina más”

- **Cómo funciona:** causa que los riñones eliminen el sodio y el agua, causando que la presión arterial baje

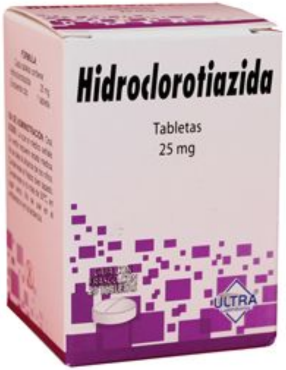


- **Dosis:** 12.5 mg al dia → 50 mg al dia

- **Efectos secundarios:** Sensibilidad de la piel al sol (fotosensibilidad), orina mucho, potasio bajo, sodio bajo, calcio alto, gota, problemas con la vista (glaucoma)

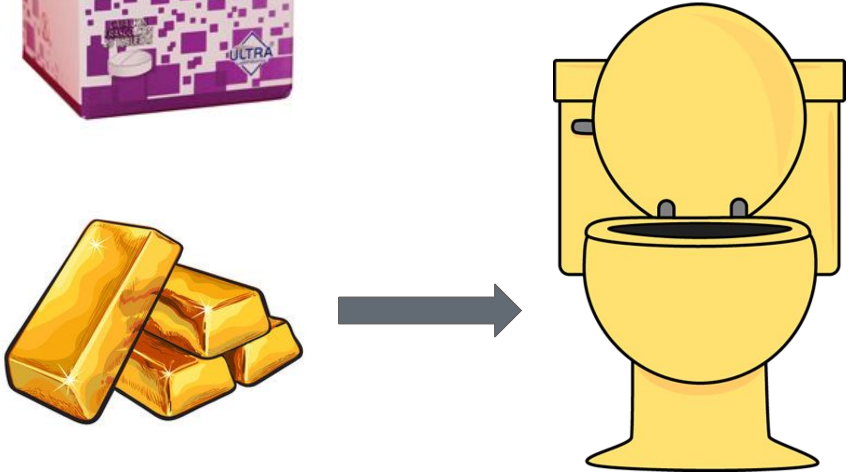

# Metoprolol (betabloqueante)

"Me **meto** a la cama porque estoy **cansado y mareado**"

- **Cómo funciona:** bloquean la hormona epinefrina - esto causa que las arterias se abran más y así ayuda a bajar la presión arterial

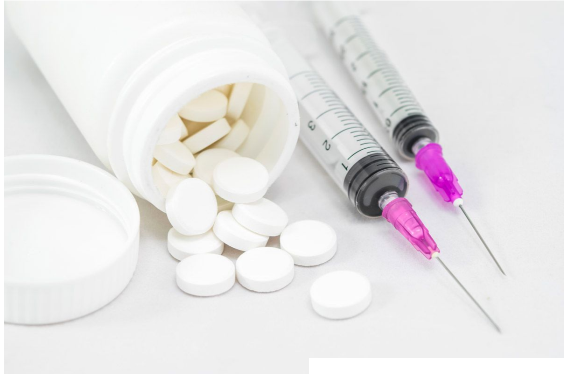

- **Dosis:** 50 mg al dia → 100 mg al dia

- **Efectos secundarios:** **pulso bajo (cansancio, mareos)**, manos o pies fríos, sibilancia, dificultad con dormir

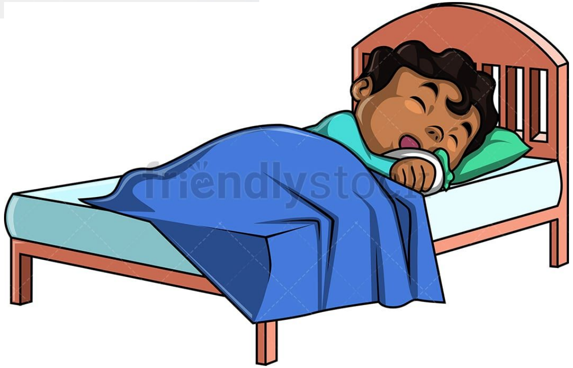

# Furosemida

"Mida su agua si no se deshidrata"

- **Cómo funciona:** causa que los riñones eliminen más sodio y agua -> baja la presión

- **Dosis:** 20 mg al dia → 600 mg al dia

- **Efectos secundarios:** Deshidratación, orina mucho, potasio bajo, sodio bajo, sarpullido, daño a los oídos

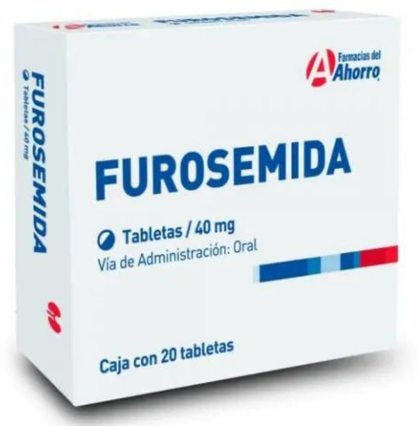


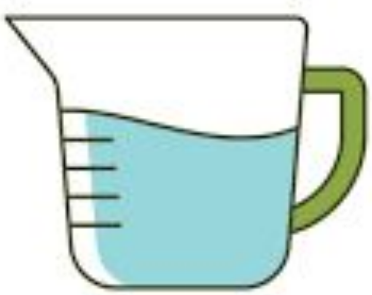

# Tratamientos Naturales - Ajo

- Estudios son muy limitados
- Puede reducir la presión en personas con hipertensión hasta 7-9 / 4-6
- Dosis: ~2 dientes de ajos por dia
- Efectos adversos
  - Dolor de estomago
  - Nausea
  - Flatulencia
- Interacciones con medicamentos
  - Anticoagulantes -> sangrado
  - Medicamentos para diabetes -> azucar baja
  - Antihipertensivo -> presión baja

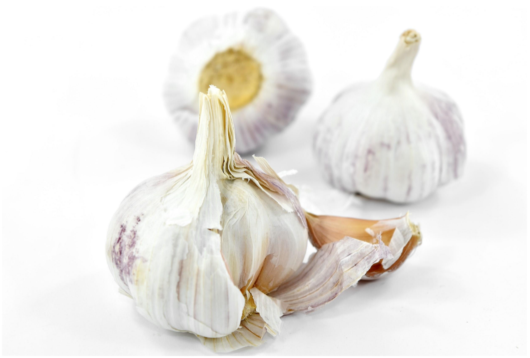

RESUMEN: Probablemente seguro y posiblemente puede ayudar bajar la presion un poco → NO DEBE REEMPLAZAR LOS MEDICAMENTOS

# Tratamientos Naturales – Vitamina C

- Importante para sanar heridas, hacer colágeno, y hacer hormonas
- Recomiendan tener 75 mg (mujeres), 90 mg (hombres) todos los días
- Presión: Posiblemente baja presión por 4/2
- Dosis: >200 mg/dia (maximo: < 2000 mg/dia)
- Efectos adversos (más común con dosis grandes)
  - Desgaste dental
  - Cansancio
  - Náusea, vómito, dolor de estómago, diarrea
  - Calculo renal
- Interacciones con medicamentos
  - En personas deficientes en Vitamina C -> estrógeno alto
  - Medicamento de la tiroides
  - Anticoagulantes -> coagulo

| Contenido de Vitamina C en 100g | |
|---|---|
| Alimento | Vitamina C (mg) |
| Guayaba | 168.4 |
| Pimiento amarillo | 155.9 |
| Perejil | 133.3 |
| Coliflor | 88.5 |
| Kiwi | 83.7 |
| Brócoli | 75.5 |
| Papaya | 61.8 |
| Fresa | 58.8 |
| Mango | 53.2 |
| Mandarina | 51.1 |
| Chícharo | 48.1 |
| Jugo de limón | 45.9 |
| Nanche | 38.4 |
| Naranja | 33.4 |

RESUMEN: Vitamina C es importante para nuestra salud y posiblemente ayuda a bajar la presión, pero no se necesita en grandes cantidades!

# Preguntas Frequentes

**Mi paciente con hipertensión empezó a tomar un medicamento para la presión, ahora tiene la presión normal. ¿Qué hacemos con su medicamento?**

¡Qué bueno! ¡Está funcionando el medicamento, siga tomándola! Cuidado con la presión baja

**A mi paciente se le olvidó tomar su medicamento en la mañana, puede tomar doble la dosis en la tarde?**

Los efectos secundarios pueden afectar a su paciente más fuerte si toma doble la dosis normal. Es mejor continuar su rutina y encontrar una manera de recordarse todos los días. Si la dosis es una vez por día, puede tomarla en la tarde.

# Pregunta

Su paciente tiene la presión elevada a 155/100. Cuando usted le sugiere empezar un medicamento, su paciente dice que prefiere no tomar medicamento y que solo quiere seguir comiendo un diente de ajo todos los días.

¿Qué le puede decir a su paciente sobre cuánto efecto tiene el ajo en la presión?

# Respuesta

- Es posible que el ajo tenga un efecto moderado en la presión
- Lo más probable es que no pueda bajar la presión a un nivel saludable con solo ajo
- Puede sugerir continuar su ajo y tal vez agregar un medicamento para ayudar a bajar la presión
- Reporte sintomas de presion baja (mareado, débil, confusión, desmayos)

# Panel de laboratorios para la hipertensión

# Panel de lípidos

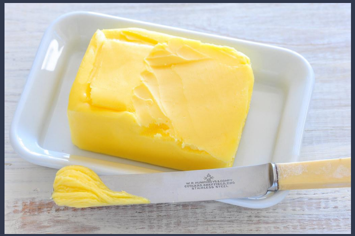

- **¿Qué es?**
  - Prueba de sangre que mide la cantidad de ciertos tipos de **lípidos (grasa)** en la sangre
- **¿Por qué es necesario?**
  - Parte de un chequeo de rutina
  - Para determinar su riesgo de enfermedad del corazón y derrame
  - Para determinar cómo su cuerpo reacciona a un medicamento

- **¿Qué mide?**
  - **HDL** - colesterol “bueno”
  - **LDL** - colesterol “malo” que se acumula en las arterias
  - **Colesterol total -** la combinación de todos los tipos de colesterol
  - **Triglicéridos** - la grasa que viene de la comida

# Cholesterol y sus arterias

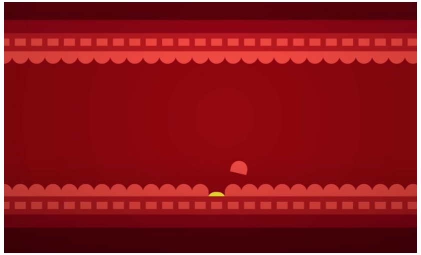

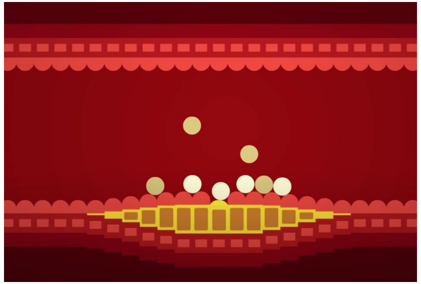

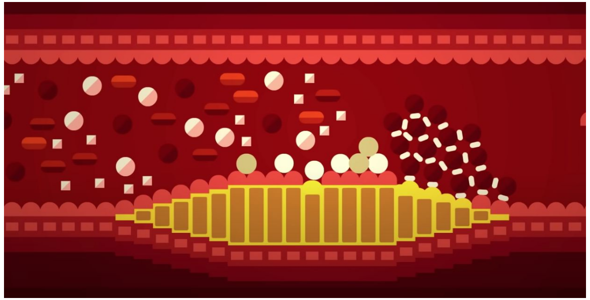

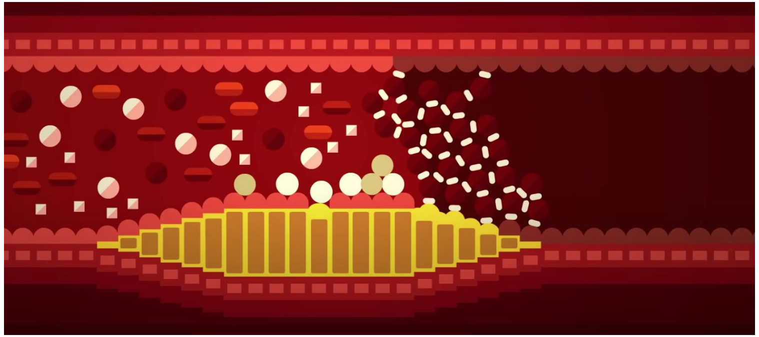

# Panel metabólico básico

- **¿Qué es?**
  - Una muestra de sangre que nos da información sobre **el metabolismo** del cuerpo
  - Se hace en ayunas

- **¿Qué vamos a medir?**
  - **Creatinina**: la función de los riñones
  - **Potasio, sodio, y cloro**: la función de los músculos, nervios y corazón y equilibrio del líquido en el cuerpo

- **¿Por qué es necesario?**
  - Para evaluar función de riñones y identificar problemas con potasio
  - Parte de un chequeo de rutina
  - Para determinar cómo su cuerpo maneja un tratamiento

# Glucosa sanguínea

- **¿Qué es?**
  - Prueba de sangre que mide los niveles de **glucosa**
  - Se hace en ayunas
- **¿Por qué es necesario?**
  - Para diagnosticar diabetes si los niveles están altos
  - Para determinar cómo su cuerpo maneja un tratamiento para diabetes

- **¿Qué mide?**
  - Los niveles de glucosa en la sangre
  - Glucosa es un tipo de azúcar que le da energía al cuerpo

# TGP (transaminasas)

- **¿Qué es?**
  - Una prueba de sangre que nos da información sobre la función de su **hígado**

- **¿Qué mide?**
  - Los niveles de TGP en la sangre pueden subir cuando hay daño al hígado

- **¿Por qué es necesario?**
  - Preparación para tomar atorvastatina
  - Para diagnosticar ciertas condiciones del hígado
  - Parte de un chequeo de rutina

# Pregunta

**Usted le está sacando sangre a un paciente para unos laboratorios.**

**Su paciente le pregunta que mide la prueba de ALT.**

**¿Qué le puede decir usted a su paciente?**

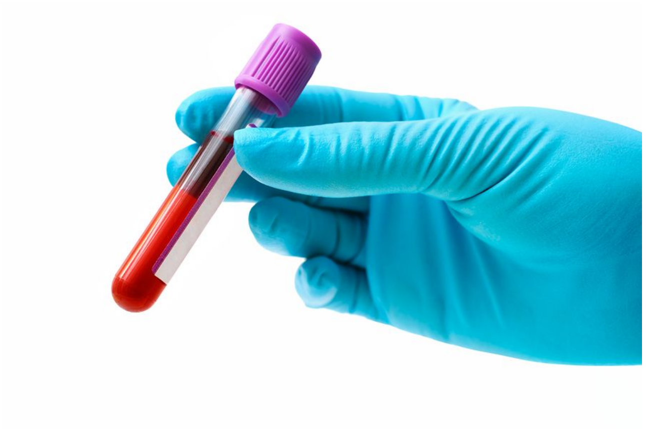

# Respuesta

- Si los niveles de ALT están altas, puede haber algún **daño al hígado**

- Se puede usar para **diagnosticar enfermedades** o como parte de un **chequeo de rutina o para monitorear medicamentos que están tomando**

# Comorbilidades

# ¿Por qué importan las comorbilidades?

- **Comorbilidad = condición o enfermedad que paciente tiene aparte de la hipertensión**
- **Puede afectar participación en el programa**
- **Afectan recomendaciones de medicamento**
  - Ejemplo: Enalapril o losartan recomendados si tiene historia de diabetes, enfermedad de corazón, o enfermedad renal
- **Afectan riesgo para complicaciones**
  - Ejemplo: Colesterol alto → más riesgo para infarto o derrame
- **Afecta seguridad de medicamentos**
  - Dosis inicial y dosis máxima de ciertas medicamentos varía con edad y comorbilidades

# Colesterol Alto (hiperlipidemia)

**¿Qué es?**

- Demasiado colesterol malo o triglicéridos en la sangre o bajo colesterol bueno en la sangre
- Sube el riesgo de ataque al corazón y derrames cerebrales

**¿Síntomas?**

- No sintomas

**¿Cómo se identifica?**

- Panel de lipidos

**¿Por qué importa?**

- En ciertos casos, se recomienda atorvastatina para bajar colesterol malo

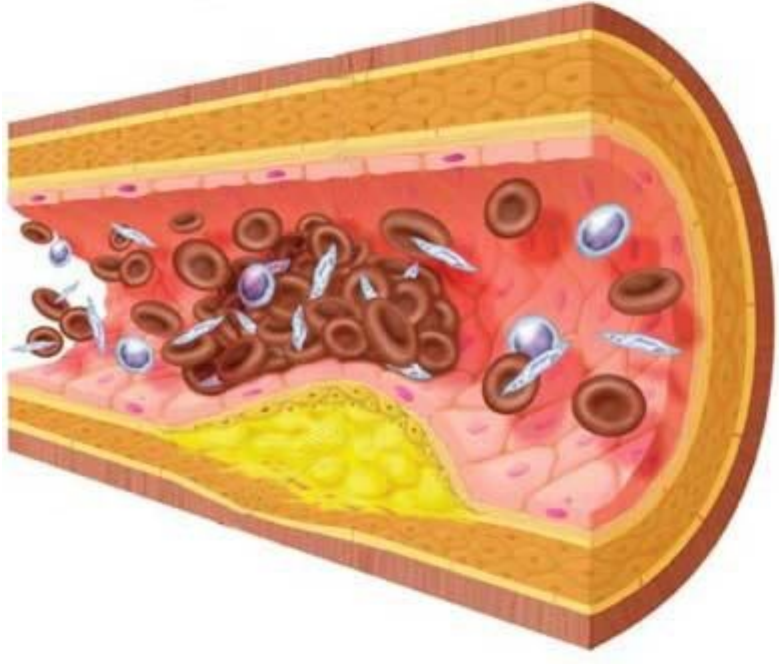

# Diabetes

**¿Qué es?**

- El cuerpo no puede absorber el azúcar efectivamente → azúcar en la sangre sube
- Sube el riesgo para ataques al corazón, derrames, hipertensión y más

**¿Síntomas?**

- Orinar mas a menudo
- Mucha sed
- Vision borrosa

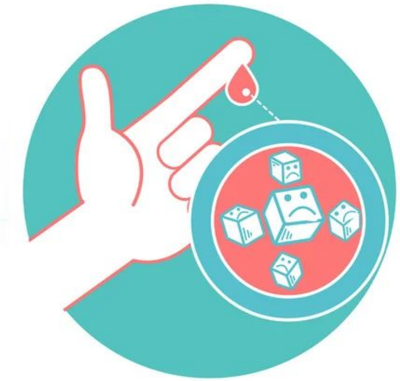

**¿Cómo se identifica?**

- Prueba de A1C
- Glucosa sanguinea

**¿Por qué importa?**

- Enalapril o losartan preferidos para manejar presión en diabéticos

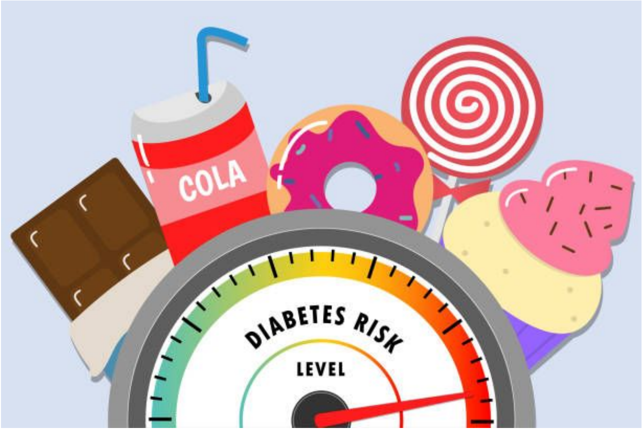

# Cirrosis (enfermedad crónico del hígado)

**¿Qué es?**

- Daño continuo al hígado → causa que el hígado se ponga duro con cicatrices
- Puede ser por un virus, alcohol o por sobrepeso

**¿Síntomas?**

- **Tinte amarillo en la piel y los ojos (ictericia)**
- Vomitar sangre
- Picazón de la piel
- Orina oscura

**¿Como se identifica?**

- Prueba de ALT y otras pruebas de sangre, ultrasonido o otros imagenes

**¿Por qué se importa?**

- No se recomienda el uso de atorvastatina si tiene enfermedad de hígado
- Se recomienda dosis más baja de amlodipina y losartan

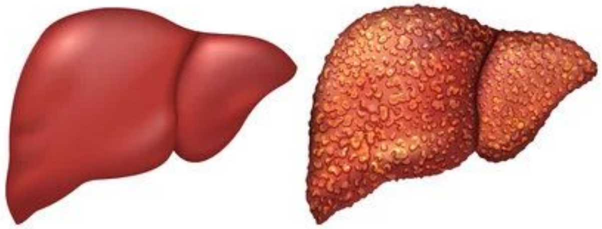

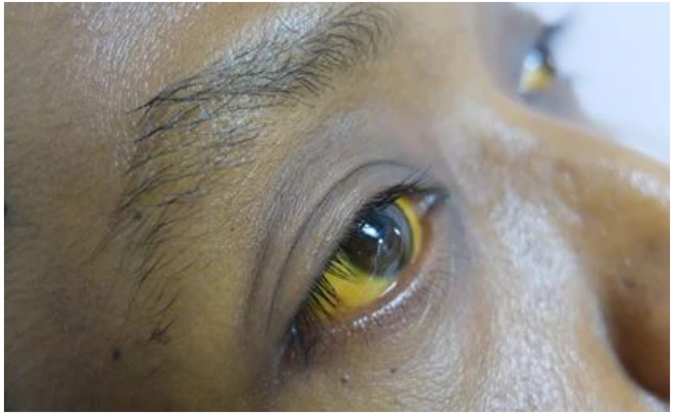

# Enfermedad crónica de los riñones

**¿Qué es?**

- Riñones están dañados y no pueden filtrar la sangre bien

**¿Síntomas?**

- Nada específico (hinchazón en las piernas, cansancio, debilidad, orina menos que normal)

**¿Cómo se identifica?**

- Creatinina

**¿Por qué importa?**

- Aumenta riesgo cardiovascular
- Enalapril o losartán pueden proteger los riñones
- Afecta la dosis de enalapril

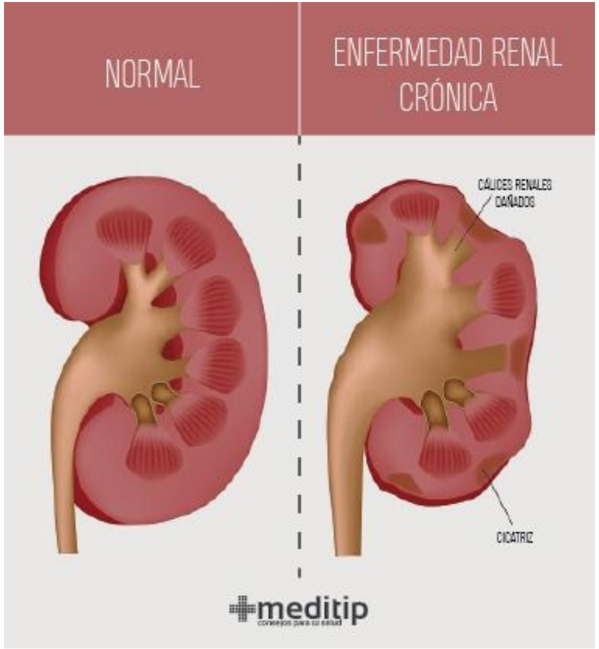

# Enfisema (EPOC)

**¿Qué es?**

- Daño dentro de los pulmones por causa de fumar o un problema genético

**¿Síntomas?**

- Falta de respiración
- Silbidos al respirar
- Tos con mucosidad

**¿Cómo se identifica?**

- Una máquina que mide cómo respira (espirómetro)
- Rayos equis

**¿Por qué importa?**

- Tener precaución con metoprolol

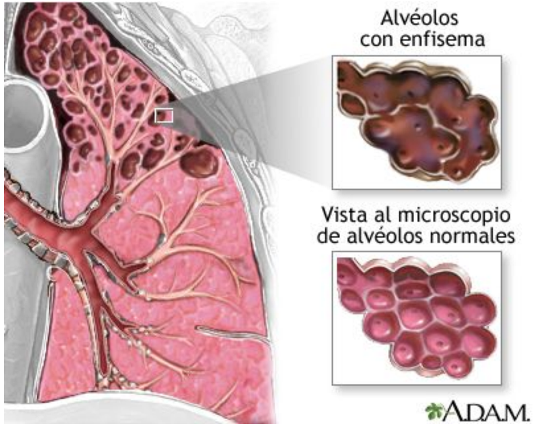


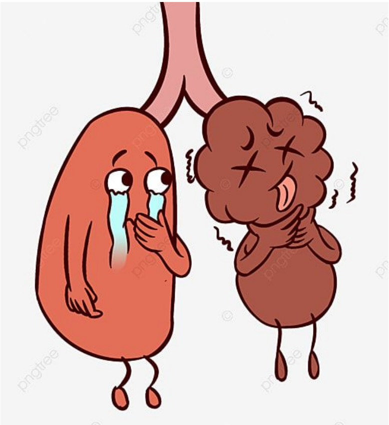

# Enfermedad de las arterias coronarias

**¿Qué es?**

- Arterias del corazón limitan la sangre y oxígeno que recibe el corazón → ataque al corazón

**¿Síntomas?**

- Muchas veces solo tiene síntomas cuando hace ejercicio
- Angina (dolor de pecho)
- Dificultad al respirar
- Dolor en el brazo izquierdo, cara, espalda o abdomen

**¿Como se identifica?**

- Prueba de estrés o angiograma
- A veces hay señales en electrocardiograma

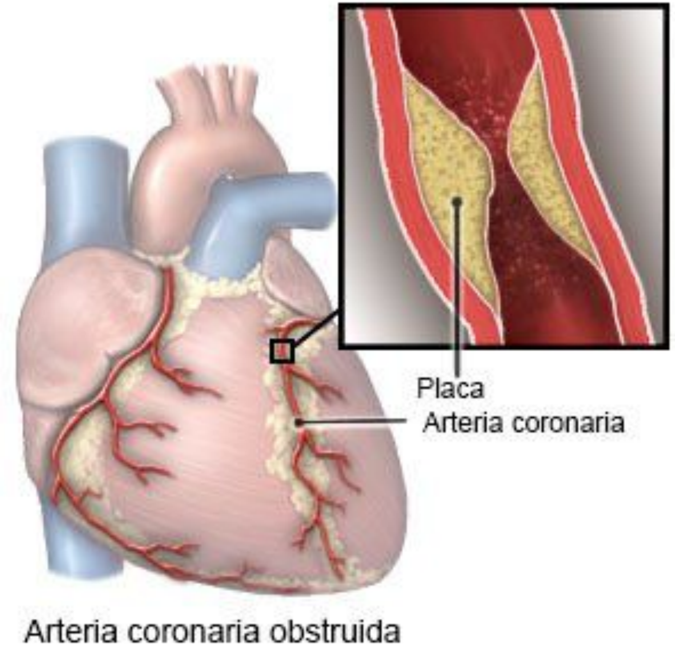

# Ataque al corazón

## ¿Qué es?

- El flujo de sangre al corazón se bloquea y parte del corazón se empieza a morir

## ¿Síntomas?

- Dolor o presión en el pecho que sigue al brazo izquierdo
- Dificultad al respirar
- Mareos y sudor

## ¿Cómo se identifica?

- Electrocardiograma y/o ecocardiograma
- Pruebas de sangre

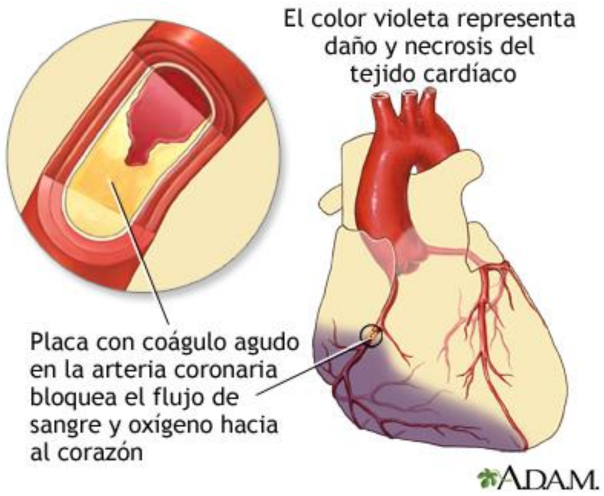

# Fallo cardiaco

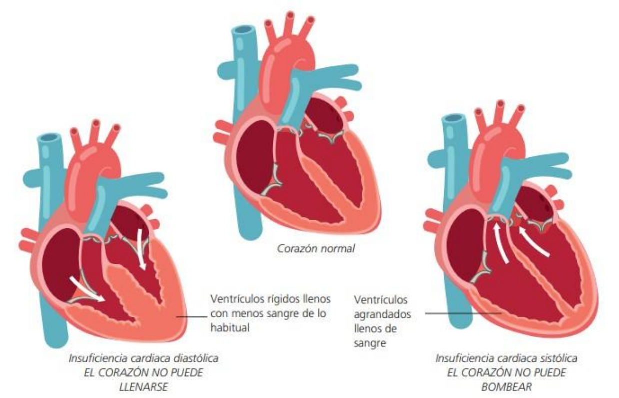


## ¿Qué es?

- Cuando el corazón no puede bombear sangre al cuerpo correctamente

## ¿Síntomas?

- No síntomas al principio
- **Hinchazón en las piernas (edema)**
- Aumento de peso rapido
- Falta de respiración

## ¿Cómo se identifica?

- Examen físico
- Prueba de BNP (sangre)
- Ecocardiograma

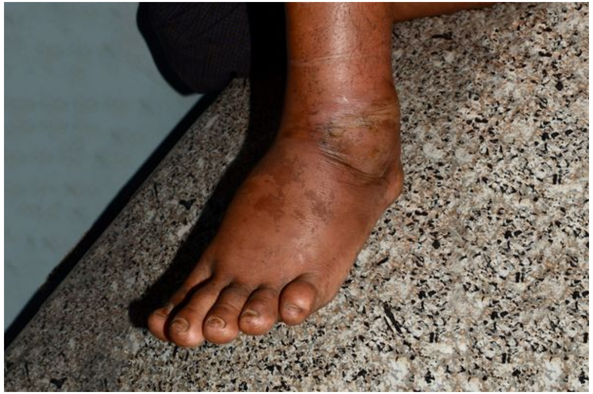

# ¿Por qué importa la enfermedad del corazón?

**Enalapril y losartan son preferidos para la hipertensión**

**Pacientes pueden necesitar otros medicamentos**

- Aspirina
- Atorvastatina
- Metoprolol
- Furosemida

**Aumenta riesgo para otros complicaciones en el futuro**

# Derrame cerebral

**¿Qué es?**

- Se detiene el flujo de sangre a parte del cerebro debido a un coágulo de sangre o una hemorragia

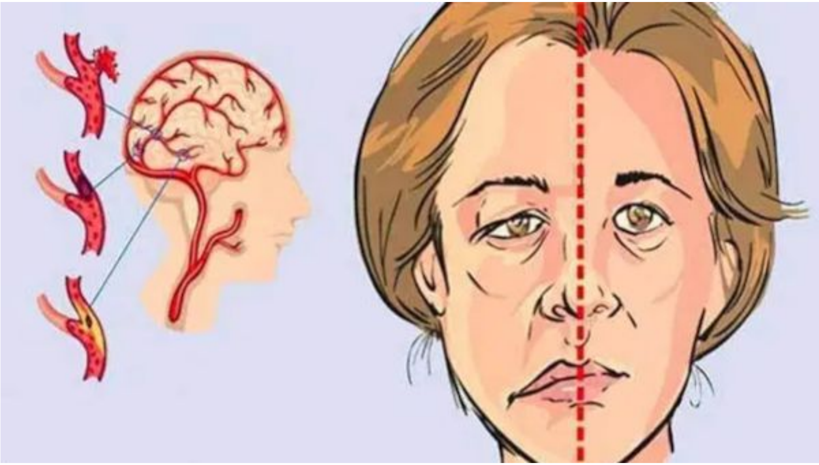

**¿Síntomas?**

- Rostro caida
- Pérdida de fuerza
- Dificultad para hablar

**¿Cómo se identifica?**

- Examen fisico, tomografia o IRM

**¿Por qué importa?**

- Control presión muy importante para prevenir otro derrame
- Paciente podría necesitar tomar aspirina y atorvastatina

# Pulso irregular

**¿Qué es?**

- Cualquier pulso fuera de lo normal
- Puede ser mas rapido, mas lento, o completamente diferente
    - Fibrilacion atrial

**¿Síntomas?**

- Palpitaciones
- Dolor de pecho
- Mareo
- Dificultad al respirar

**¿Como se identifica?**

- Examen físico
- Electrocardiograma

**¿Por qué importa?**

- Paciente podría necesitar metoprolol o otro medicamento para controlar el pulso y aspirina para prevenir derrame

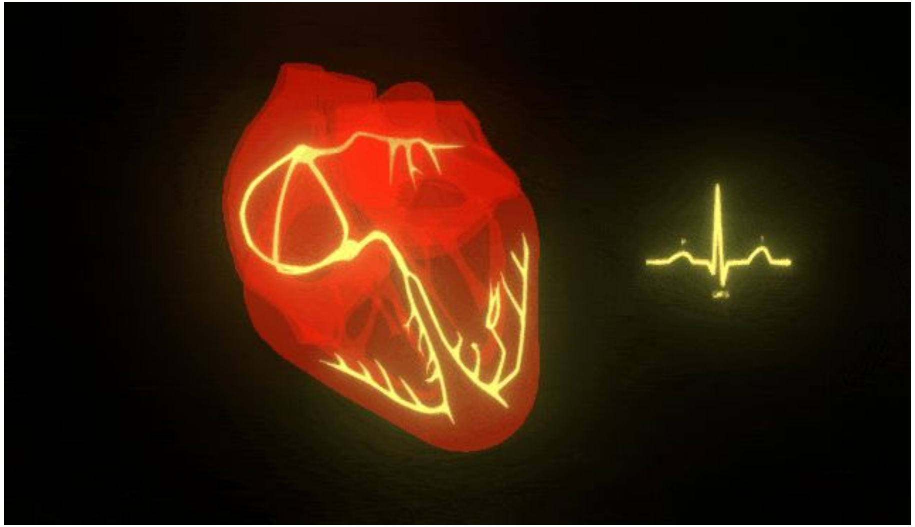

# Cáncer

**¿Qué es?**

- Células del cuerpo se dividen sin control

**¿Síntomas?**

- Depende en qué parte del cuerpo está afectado
- Ejemplo: cuello de la matriz → sangrado, dolor pélvico
- Síntomas generales → cansancio, pérdida de peso

**¿Cómo se identifica?**

- Biopsia
- Imagenes

**¿Por qué importa?**

- Si paciente tiene cáncer activa, puede afectar su participación en el programa

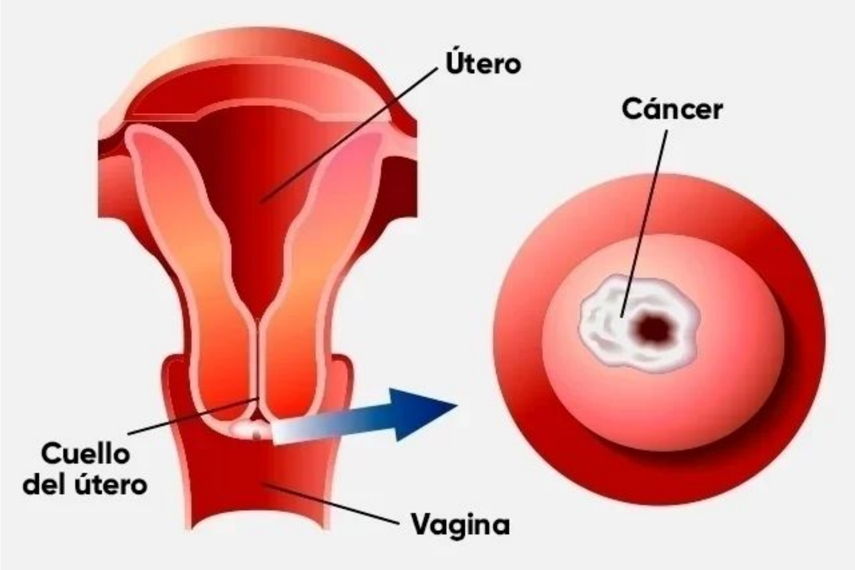

# Oxímetro de pulso

# Lo Básico

## ¿Qué mide?

- **% SpO2** → la concentración de oxígeno en la sangre
- **PR** → el ritmo cardiaco (pulso)
- **Pletismografía de pulso** → el patrón del latido del corazón

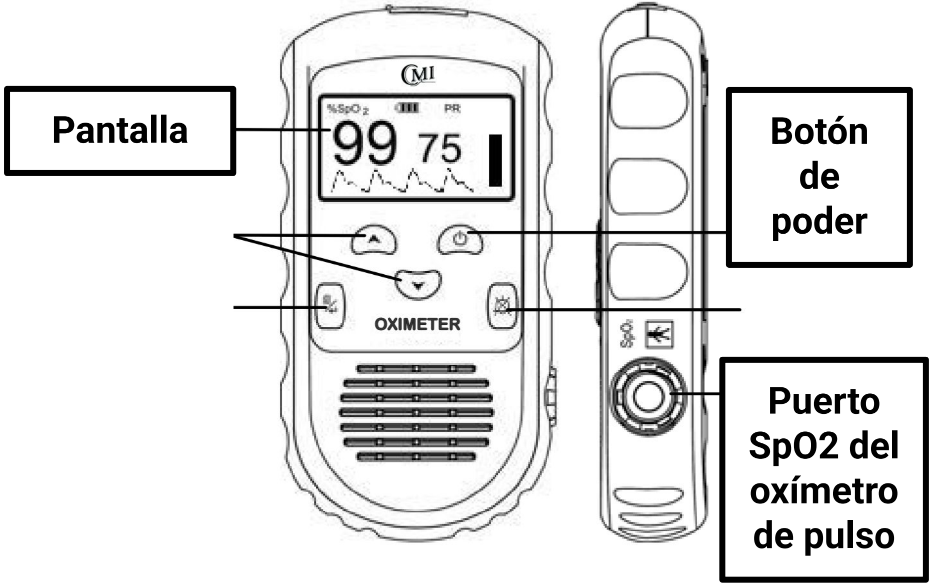

# ¿Por qué es importante?

- Si estamos preocupados que el paciente está sufriendo de **una complicación de la hipertensión o que tiene otro problema médico**
  - Ataque al corazón
  - Enfisema (EPOC)
  - Fallo cardiaco
  - Arritmia

# Pantalla principal

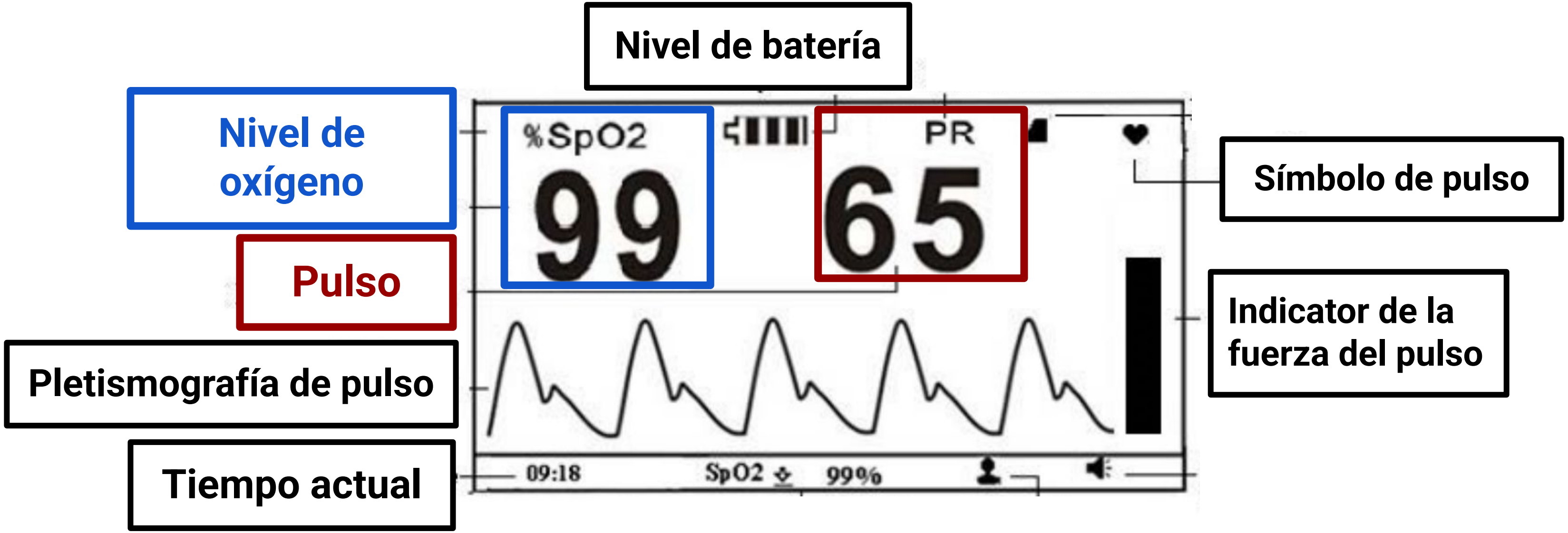

# Como se usa – parte 1

1. **Encenderlo**
   - Oprima el botón de poder para encender el oxímetro por 2 segundos
2. **Conectar el medidor de oxígeno a la máquina**
   - Conecte un lado del cable al puerto del oxímetro de pulso (en el lado)
   - Conecte el otro lado del cable al puerto del sensor de SpO2 (lo que va en el dedo)
3. **Poner el dedo**
   - Ponga el dedo índice del paciente en el medidor de oxígeno (la uña debe estar hacia arriba)

1.

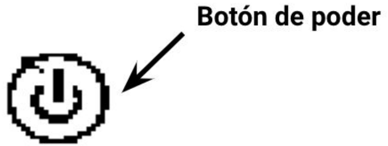


2.

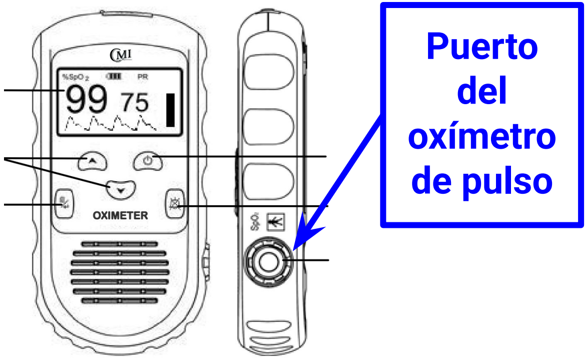


3.

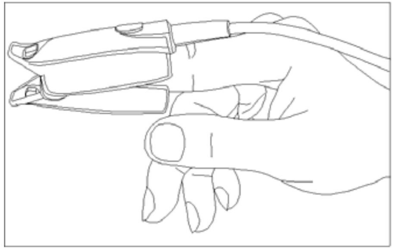

# Como se usa – parte 2

4. **Esperar**
    - Espere hasta que aparezcan 2 números en la pantalla
5. **Anotar los datos**
    - Anote el nivel de oxígeno y el pulso del paciente en la aplicación de CommCare
6. **Apagarlo**
    - Cuando termine, presione el botón de poder hasta que se apague el oxímetro de pulso

**4-5.**

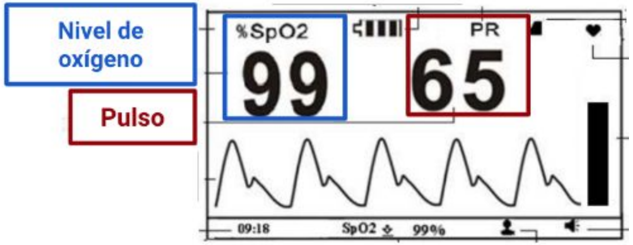


**6.**

**Botón de poder**

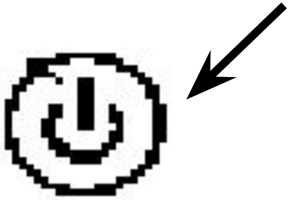

# Guía de niveles de oxígeno

## 89% o menos

- EMERGENCIA → nivel demasiado bajo que puede indicar un problema médico grave
- Contacte a su coordinador inmediatamente

## 90% a 94%

- Nivel bajo que puede indicar un problema médico pero puede ser normal
- No se necesita hacer nada por ahora

## 95%-100%

- Nivel normal

# Guía de la pletismografía de pulso

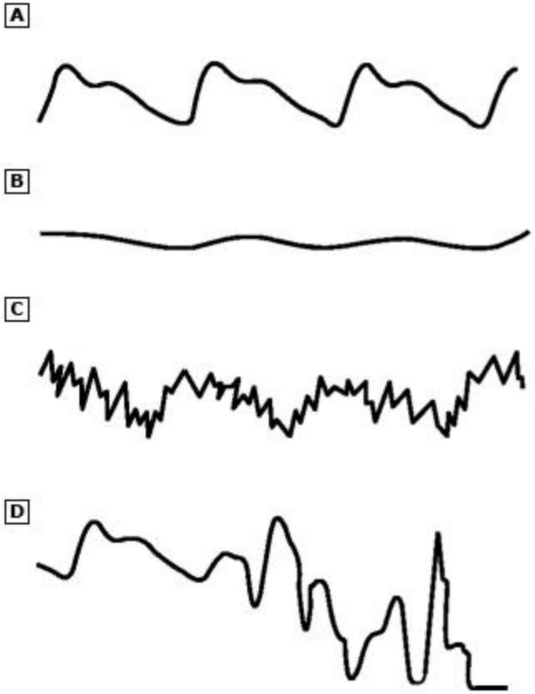


**Ritmo cardiaco normal y señal fuerte**

**Baja circulación o sensor puesto incorrectamente**

**Algo molestando al sensor**

**El paciente se está moviendo**

# ¡Gracias!

¿Preguntas?
