# Supplementary material for: Community Health Worker Feedback on an mHealth Intervention for Hypertension in Rural Guatemala: Mixed Methods Formative Study
Source: JMIR Form Res. 2026 Apr 17;10:e75471. doi: 10.2196/75471 (PMC13135166; doi:10.2196/75471)
Supplement: Multimedia Appendix 3 [file formative_v10i1e75471_app3.pdf]

Hypertension Application Questionnaire

Name ____ Age _____________ Gender ___________________

What is the highest level of education you have completed? ⬜ None ⬜ Primary ⬜ Basic ⬜ Diversified ⬜ High School ⬜ University

How often do you use a touchscreen phone (smartphone) or a tablet in your work and daily life?

⬜ Every Day ⬜ Several times per week ⬜ Once a week ⬜ A few times per month ⬜ Once a month or less

How many times have you used the hypertension application?

⬜ Less than 5 ⬜ 5 to 10 ⬜ 11 to 15 ⬜ 16 to 20 ⬜ More than 20

	Strongly disagree	Disagree	Neither agree nor disagree	Agree	Strongly Agree
I think I would like to use the application frequently.	1	2	3	4	5
I think the application is unnecessarily complicated.	1	2	3	4	5
I think the application is easy to use.	1	2	3	4	5
I think I would need the help of a technician to use this application.	1	2	3	4	5
I think the different functions of the application are well integrated.	1	2	3	4	5
I think the application is confusing.	1	2	3	4	5
I imagine that most people would learn to use this application very quickly.	1	2	3	4	5
I think the application is very difficult to use.	1	2	3	4	5
I feel very confident using the application.	1	2	3	4	5
I needed to learn many things about the application before I could use it.	1	2	3	4	5

Written Questions:

- What do you like most about the application?
- How can we improve or change the application?
- What do you like most about the hypertension program?
- How can we improve or change the hypertension program?
- What do you like most about the hypertension training?
- What would you like to improve about the hypertension training?
