# Supplementary material for: Community Health Worker Feedback on an mHealth Intervention for Hypertension in Rural Guatemala: Mixed Methods Formative Study
Source: JMIR Form Res. 2026 Apr 17;10:e75471. doi: 10.2196/75471 (PMC13135166; doi:10.2196/75471)
Supplement: Multimedia Appendix 4 [file formative_v10i1e75471_app4.pdf]

Focus Group Interview Question Guide

1. What is your overall impression of the hypertension program?
2. What is the best aspect of the program?
3. What part of the program could be improved?
4. How do you feel about working on the hypertension project?
5. What is your opinion of the training process for the hypertension project, and how have the trainings helped you?
6. What was the most challenging part of communicating with hypertension patients? For example, was it counting pills, educating patients about their medications, or managing discussions about symptoms?
7. What was the most difficult part of using the app?
8. What do you think will be the main challenges in scaling the program to hundreds of patients (up to 200), and what should we focus on?
9. What questions should we ask patients about the program?
10. Is there any area in which you would like additional training?
11. If participants are not providing specific answers, we will walk through the entire app and ask for feedback as we proceed.
